# Development of brain networks for social functions: Confirmatory analyses in a large open source dataset

**DOI:** 10.1016/j.dcn.2018.11.002

**Published:** 2018-11-20

**Authors:** Hilary Richardson

**Affiliations:** Massachusetts Institute of Technology, Department of Brain and Cognitive Sciences, 43 Vassar Street, 46-4021, Cambridge, MA, 02139, United States

**Keywords:** Theory of mind, Functional connectivity, Resting state, Development, fMRI, Open source data

## Abstract

Human observers show robust activity in distinct brain networks during movie-viewing. For example, scenes that emphasize characters’ thoughts evoke activity in the “Theory of Mind” (ToM) network, whereas scenes that emphasize characters’ bodily sensations evoke activity in the “Pain Matrix.” A prior exploratory fMRI study used a naturalistic movie-viewing stimulus to study the developmental origins of this functional dissociation, and the links between cortical and cognitive changes in children’s social development (Richardson et al., 2018). To replicate and extend this work, the current study utilized a large publicly available dataset (n = 241, ages 5–20 years) (Alexander et al., 2017) who viewed “The Present” (Frey, 2014) and completed a resting state scan (n = 200) while undergoing fMRI. This study provides confirmatory evidence that 1) ToM and pain networks are functionally dissociated early in development, 2) selectivity increases with age, and in ToM regions, with a behavioral index of social reasoning. Additionally, while inter-region correlations are similar when measured during the movie and at rest, only inter-region correlations measured during movie-viewing correlated with functional maturity. This study demonstrates the scientific benefits of open source data in developmental cognitive neuroscience, and provides insight into the relationship between functional and intrinsic properties of the developing brain.

## Highlights

• Confirmatory evidence for developmental change in social brain regions.
• Replication of a neural correlate of social reasoning in a novel movie paradigm.
• Network properties during movie, but not rest, are related to functional response.
• Movie experiments are promising for large, replicable studies of brain development.

## 1 Introduction

The adult brain is comprised of functional networks of brain regions that are commonly engaged in particular cognitive tasks (Kanwisher, 2010). The characterization of these functional networks in the adult brain has paved the way for pediatric studies that aim to understand the developmental origins of this functional dissociation, and to link neural changes within these networks to cognitive development in childhood. Discovering robust neural markers of cognitive development could provide specific targets for cognitive and clinical interventions. However, a key challenge for this endeavor is to study the properties of functional networks in children.

Naturalistic movie-viewing experiments may provide a solution to this challenge. While functionally selective regions have traditionally been studied via experiments that measure responses across multiple trials of experimental and control conditions, movie stimuli can be tailored in length, are engaging for young children, and minimize participant motion (Cantlon and Li, 2013; Vanderwal et al., 2015). Movie-viewing experiments require minimal instruction, and are easily replicable across research sites. Accumulating evidence from fMRI studies of adults suggests that movie-viewing experiments can be used to replicate functional divisions in cortex traditionally characterized with lengthy, well-controlled experiments (Hasson, 2004; Hasson et al., 2010; Wagner et al., 2016). For example, Jacoby et al. (2016) used a <6 min movie (Disney Pixar’s “Partly Cloudy” (Reher and Sohn, 2009)) to localize two functionally dissociated cortical networks for reasoning about distinct aspects of other people (Jacoby et al., 2016). One network, comprised of bilateral temporoparietal junction (TPJ), precuneus, and medial prefrontal cortex (e.g., Saxe and Kanwisher, 2003), is recruited to reason about others’ *minds* (the “Theory of Mind” (ToM) network), while a second network, comprised of bilateral insula, medial frontal gyrus, and secondary sensory motor cortex, and dorsal anterior middle cingulate cortex (e.g. Zaki et al., 2016), is recruited to reason about others’ *bodies* (the “Pain Matrix”).

The functional dissociation between the ToM and Pain networks has been studied across a wide range of social and experimental contexts. For example, when considering others’ actions, the ToM network is recruited to reason about *why* it was performed (with what intention), while the Pain Matrix is recruited to reason about *how* it was performed (with what muscle movements) (Spunt et al., 2015). When considering others’ painful experiences, the ToM network is recruited to reason about *emotional pain* (negative emotions like grief or sadness), while the Pain Matrix is recruited to reason about *physical pain* (i.e., the bodily experience itself) (Bruneau et al., 2012, 2013). When viewing others’ suffering, (part of) the ToM network is recruited when observers feel care and concern towards the other, while the Pain Matrix is recruited when observers experience vicarious or shared negative feelings (Ashar et al., 2017; Singer and Klimecki, 2014). Thus, across different experimental paradigms, labs, and social contexts, ToM and Pain networks are recruited to reason about different kinds of *internal states*: the internal states of others’ minds (e.g., beliefs, desires (Gallagher et al., 2000; Saxe and Kanwisher, 2003), and emotions (regardless of valence) (Koster-Hale et al., 2017; Skerry and Saxe, 2015)), and the internal states of others’ bodies (e.g., muscle movements (Spunt et al., 2015), sneezes (Lombardo et al., 2010), and pain (Bruneau et al., 2012)). The movie stimulus used by Jacoby et al. (2016) (“Partly Cloudy” (Reher and Sohn, 2009)) is particularly useful for studying responses in the ToM and Pain networks because it includes scenes that emphasize characters’ beliefs, desires, and emotions, and scenes that emphasize characters’ bodily states and physical pain. Additionally, the content and length of this movie make it an ideal experimental paradigm for measuring functional responses in young children.

Indeed, a recent exploratory fMRI study of children as young as three years old (n = 122, 3–12 years) measured functional responses in ToM and Pain brain regions while they viewed “Partly Cloudy” (Richardson et al., 2018). A key result of this study was that signatures of the cortical division between ToM and pain brain regions were present in three-year-old children: responses in ToM brain regions were more correlated with other ToM regions than with regions in the Pain Matrix (and vice versa), and responses in three-year-old children in each network were significantly correlated with the average adult timecourse. This study also found significant developmental change in inter-region correlations and functional maturity throughout childhood, suggesting continued development and refinement of the functional responses in both networks. The responses in ToM and Pain networks became increasingly distinct (anti-correlated) over childhood. While three-year-old timecourses looked similar to those of adults, some ToM scenes evoked responses in the Pain Matrix, and some pain scenes evoked responses in the ToM network. Across all children, responses to a particular ToM scene were positively correlated with cognitive performance on an independent behavioral test of ToM (https://osf.io/g5zpv/). If robust, such a neural marker could be a useful index for designing and evaluating interventions aimed to improve social cognitive abilities.

This prior study provided insights into the development of the cortical division between ToM and pain networks, and suggested a plausible neural marker of social cognitive behavior. However, this study had two key limitations. First, it is critical to use confirmatory analyses to ensure that the results of exploratory analyses are robust and replicable. Developing generalizable neural markers of social cognitive behaviors is important for developing and testing the effectiveness of social cognitive training paradigms and clinical interventions. The second limitation concerns the link between network properties and functional responses. Specifically, the prior study found that the extent to which ToM and Pain brain regions were functionally dissociated, as measured via inter-region correlations within and between the two networks (i.e., how similar the response in one region is to that of another region), correlated with the functional maturity of the response (i.e., how similar the response was to the average adult response). Inter-region correlations are considered to measure functional or “effective” connectivity (Blank et al., 2014; Hasson, 2004; Hasson et al., 2010), but could be driven by functional response profiles (e.g., two regions activate and deactivate to the same content within the movie stimulus), or could reflect intrinsic network properties that are present at rest, i.e., in absence of stimuli (Fox et al., 2005; Greicius et al., 2003) (e.g., two regions activate and deactivate together regardless of stimulus). Intrinsic networks largely correspond to the functional divisions in cortex: brain regions that are correlated during cognitive tasks are also correlated at rest (Cole et al., 2014; Greicius et al., 2003; Miall and Robertson, 2006).

Because inter-region correlations were measured during movie-viewing, the prior study could not determine the relative contributions of functional or intrinsic properties to inter-region correlations, and therefore could not determine the nature of the link between functional maturity and inter-region correlations. One hypothesis is that the relationship between functional maturity and inter-region correlations is driven by the *stimulus driven response* in these two networks during movie-viewing. That is, systematic functional responses to stimuli organize these brain regions into two functionally distinct, anti-correlated networks. Inter-region correlations during functional tasks could subsequently shape intrinsic inter-region correlations. Previous work has found that stimulus-elicited connectivity predicts resting state connectivity patterns longitudinally (Gabard-Durnam et al., 2016), and resting state connectivity can be altered via intensive exposure to particular cognitive tasks (Mackey et al., 2013). Thus, engaging specific brain regions via functionally specific tasks could drive regions within a network to become correlated with one another, and anti-correlated with regions in other networks, and these functional dissociations may influence the intrinsic connectivity between brain regions at rest. Alternatively, this relationship could be driven by the *intrinsic properties* of ToM and pain networks. Intrinsic networks are apparent by the end of the first year of life (Yeo et al., 2011), if not earlier (van den Heuvel and Thomason, 2016), and become more distinct over childhood (Chai et al., 2014). Development of intrinsic networks could plausibly precede and influence the emergence of systematic functional responses. Of course, functional responses could be similarly early developing, but there is much less functional MRI data from infants and young children, due to the methodological challenges of scanning these populations while awake (though see Deen et al., 2017).

In order to address these limitations, the current study was conducted with two goals: (1) to use confirmatory analyses to replicate the results of prior exploratory analyses, and (2) to characterize the nature of the link between stimulus-driven responses and inter-region correlations within and between the ToM and Pain networks during movie-viewing. A large, publicly available dataset of five to twenty year olds (n = 241) (Alexander et al., 2017) who viewed Jacob Frey’s “The Present” (2014) while undergoing fMRI was analyzed using methods identical to the prior study. Parents or guardians of participants completed a behavioral metric of social reasoning: the Social Communication Questionnaire (SCQ (Rutter et al., 2003)). Confirmatory evidence strengthens the confidence in results based on exploratory analyses, and in this case, tests the generalizability of the results to a more heterogeneous sample of participants and under a new experimental context (i.e., a different movie paradigm and behavioral measure of social reasoning). A large subset of the participants (n = 200) additionally completed a resting state scan, enabling the current study to help clarify the link between the development of stimulus-driven functional responses and inter-region correlations. While the current cross-sectional study cannot determine predictive relationships or causal order of development between intrinsic and functional networks, it can test whether the functional maturity of responses in ToM and pain brain regions are specifically related to inter-region correlations during movie-viewing, or are more generally related to inter-region correlations that are intrinsic, i.e., present at rest.

## 2 Methods

### 2.1 Participants

Participants were a subset of participants recruited by the Child Mind Institute (Alexander et al., 2017). The final sample included 241 participants, 200 of whom additionally had usable resting state data. Participants who completed both “The Present” in addition to an anatomical (T1) scan were downloaded from Data Releases 1.1 and 2.1 (n = 322); 314 of these participants additionally completed a resting state scan. Participants were excluded from analyses for excessive motion during the scan (Present: n = 7; Rest: n = 45) or failed registration/lack of sufficient coverage (Present: n = 74; Rest: n = 66, see fMRI Data Analysis for detailed exclusion criterion). For inter-region correlation analyses, three additional participants were subsequently excluded for having outlier correlation values (see Inter-region Correlation section of Methods), leaving n = 238 (“Present”) and n = 200 (Resting) participants for these analyses.

In order to make comparisons between this sample and a previous study (Richardson et al., 2018), some analyses were conducted specifically in children (n = 186 5–12 year old participants; M(SD) age: 9.1(2.1) years, 60 females, n = 153 right-handed; resting state subset: n = 151, M(SD) age: 9.3(2.1) years, 50 females, n = 124 right-handed) and adolescents/young adults (n = 55 13–20 year old participants: M(SD) age: 15.3(1.9) years, 26 females, n = 49 right-handed; resting state subset: n = 51, M(SD) age: 15.4(1.8) years, 25 females, n = 45 right-handed), separately. A low/matched motion subset of participants was created in order to directly compare response timecourses during “The Present” to those at rest (children (ages 5–12 years): n = 81, M(SD) age: 9.4(2.1), 30 females; full sample (ages 5–19 years: n = 106, M(SD) age: 10.8(3.1) years, 45 females; see Methods for more details about the creation of this subset). The low/matched motion subset is thus slightly older and includes disproportionately fewer male participants relative to the “full” sample; these variables were not statistically different between samples (ps>.2).

All participants were recruited by the Child Mind Institute (CMI) via a community-referred recruitment model (Alexander et al., 2017). Relative to the prior sample, the sample recruited by the CMI appears to be more representative in terms of non-verbal IQ (see Supplementary Fig. 1). Additionally, unlike the prior study (Richardson et al., 2018), which recruited participants with no known cognitive or neural disorders, many of the participants of the current study had or received clinical diagnoses at the time of testing. The most common diagnosis among participants was Attention-Deficit/Hyperactivity Disorder; see Supplemental Materials for visualizations of clinical diagnoses (Supplementary Fig. 2) and for a discussion of subject attrition. All adult participants gave written consent; parent/guardian consent and child assent was received for all child participants. The Chesapeake Institutional Review Board approved recruitment and experiment protocols; the Committee on the Use of Humans as Experimental Subjects (COUHES) at the Massachusetts Institute of Technology and the Child Mind Institute approved data access and analyses.

### 2.2 FMRI stimuli

During the functional MRI scan, participants watched Jacob Frey’s “The Present” (2014), a 3.5-minute animated movie (https://vimeo.com/152985022). During the resting state scan, participants were instructed to keep their eyes open and fixate on a crosshair in the middle of the screen. The resting state scan was completed prior to the functional movie-viewing task.

### 2.3 FMRI data acquisition

Prior to the scan, participants completed a mock scan in order to become acclimated to the scanner environment, and to learn how to stay still.

Whole-brain structural and functional MRI data were acquired on a 3-Tesla Siemens Tim Trio scanner located at the Rutgers University Brain Imaging Center, using the standard Siemens 32-channel head coil and CMRR simultaneous multi-slice echo planar imaging sequence. T1-weighted structural images were collected in 224 sagittal slices with 0.8 mm isotropic voxels (%FOV Phase: 100%). Functional data were collected with a gradient-echo EPI sequence sensitive to Blood Oxygen Level Dependent (BOLD) contrast in 60 slices covering the whole brain (TR: 800 ms, TE: 30 ms, flip angle: 31**°**, multi-band acceleration: 6). Functional data during “The Present” were acquired in a single 3.5-minute run (250 volumes); resting state data were collected across two 5.1-minute runs (375 volumes per run). Primary analyses of resting state data were conducted on the first 250 volumes, in order to match amount of data across scan type.

### 2.4 FMRI data analysis

FMRI data were analyzed using SPM8 (http://www.fil.ion.ucl.ac.uk/spm) (Friston, 1994) and custom software written in Matlab and R, using identical procedures to those used in the study that was the target for replication (Richardson et al., 2018). Functional images were registered to the first image of the run; that image was registered to each participant’s anatomical image, and each participant’s anatomical image was normalized to the Montreal Neurological Institute (MNI) template. Registration of each individual’s brain to the MNI template was visually inspected, including checking the match of the cortical envelope and internal features like the AC-PC and major sulci. All data were smoothed using a Gaussian filter (5 mm kernel).

Artifact timepoints were identified via the ART toolbox (https://www.nitrc.org/projects/artifact_detect/) (Whitfield-Gabrieli et al., 2011) as timepoints for which there was 1) more than 2 mm composite motion relative to the previous timepoint or 2) a fluctuation in global signal that exceeded a threshold of three standard deviations from the mean global signal. Data were excluded from analyses if one-third or more of the timepoints collected (per scan type) were identified as artifact timepoints (Present: 83 TRs, n = 7 participants excluded; Resting: 250 TRs, n = 43 participants excluded; n = 2 additional participants excluded for >83 TRs motion in truncated Resting scan). For subsequent analyses of the resting state scan, only the first 250 TRs were used, in order to match the amount of data analyzed across tasks. The number of motion artifact timepoints was included as a covariate in all analyses. In the current dataset, number of artifact timepoints was highly correlated with mean translation during both scans (*r*s>.62; ps<2.2 × 10^−12^). Because this measure was not normally distributed (ps<3.5 × 10^-16^), spearman correlations were used when including amount of motion as a covariate in partial correlations. Number of artifact timepoints (henceforth, “Motion”) during “The Present” decreased significantly with age in the full sample (Child (n = 186): M(SD) = 14.6(15), Adolescents/Young Adults (n = 55): M(SD) = 9.0(9.4), linear regression on motion: effect of age (continuous): b=-0.21, t=-3.2, p = .001); motion during the truncated resting state scan decreased marginally with age in the full sample (Child (n = 151) M(SD) = 10.3(20.4), A/YA (n = 51) M(SD) = 8.3(24.8), linear regression on motion: effect of age (continuous): b= −.13, t=−1.9, p = .06). Among 5–12 year old children, motion was significantly negatively correlated with age in the resting state scan, but not during the “Present” (spearman correlation test: Present: *r*_s_(184)=−.11, p = .13; Resting: *r*_s_(149)=−.17, p = .04). SCQ score was not correlated with motion during either scan (spearman correlation test: Present: *r_s_*=−.05, p = .51; Resting: *r_s_*=−.01, p = .88). See Supplementary Figure 6 for a visualization of the amount of motion in this sample.

A low- and matched-motion subset of participants (n = 106 participants, including n = 81 5–12 year old children) was used to directly compare inter-region correlations during “The Present” and at rest. Participants were first selected for inclusion in this subset if they had fewer than 10% of timepoints identified as motion artifact in both scans (<25 timepoints). Participants were subsequently excluded based on the difference in motion between the two scans, until a motion-matched sample was obtained (two-tailed paired *t*-test on number of artifact timepoints: children: t(80)=−.46, p = .64; full sample: t(105)=−.34, p = .74). Finally, because this sample was specifically created to test for significant task-by-age interactions on inter-region correlations, the four oldest participants with the largest difference in motion between the two scans were excluded, such that the task-by-age interaction on amount of motion was non-significant (children: NS effect of task-by-age interaction: p = .12; regression on motion without interaction: NS effect of task: p = .65, NS effect of age: p = .07; full sample: NS effect of task-by-age interaction: p = .18; regression on motion without interaction: NS effect of task: p = .74, effect of age: p = .001).

Region of interest (ROI) analyses were conducted using group ROIs. ToM and Pain Matrix group ROIs were created in an independent group of adults (n = 20), scanned by Evelina Fedorenko and colleagues, as previously described (Richardson et al., 2018). These group ROIs were used for easy comparison to the previous study. Note that while Pain Matrix ROIs are referred to as such, prior studies suggest that these regions are recruited when adults and children think about bodily sensations other than/in addition to physical pain (e.g., muscle movements (Spunt et al., 2015), sneezes (Lombardo et al., 2010)).

All timecourse analyses were conducted by extracting the scaled, preprocessed timecourse from each voxel per group ROI. Nearest neighbor interpolation over artifact timepoints was applied (for methodological considerations on interpolating over artifacts before applying temporal filters, see Carp, 2013; Hallquist et al., 2013), and two kinds of nuisance covariates were regressed out in order to reduce the influence of motion artifacts: 1) motion artifact timepoints, and 2) five principle component analysis (PCA)-based noise regressors generated using CompCor within individual subject white matter masks (Behzadi et al., 2007). White matter masks were eroded by two voxels in each direction, in order to avoid partial voluming with cortex. CompCor regressors were defined using scrubbed data (i.e. artifact timepoints were identified and interpolated over prior to running CompCor). The residual timecourses were then high-pass filtered with a cutoff of 100 s. Timecourses from all voxels within an ROI were averaged, creating one timecourse per group ROI, and artifact timepoints were subsequently excluded (NaNed).

#### 2.4.1 Inter-region correlation analyses

In inter-region correlation analyses, each ROI timecourse (excluding the first three timepoints) was correlated with every other ROI’s timecourse, per subject, and these correlation values were Fisher z-transformed. Within-ToM correlations were the average correlation from each ToM ROI to every other ToM ROI, within-Pain correlations were the average correlation from each Pain ROI to every other Pain ROI, and across-network correlations were the average correlation from each ToM ROI to each Pain ROI. Based on the previous study, a range of expected values for inter-region correlations was calculated as the average within-ToM, within-Pain, and across-ToM Pain correlation in the 5–12 year old and adult participants from the original study, plus or minus three standard deviations (wi-ToM: −.03 – .83; wi-Pain: −.05 – .75; ac-ToM-Pain: −.55 – .51). Adults as well as 5–12 year old children were included in this calculation in order to better suit the current sample (ages 5–20 years old). Data points outside of this range were considered outliers and excluded from inter-region correlation analyses (Present: n = 3; Resting: n = 11). During both types of scans, within-ToM correlations were normally distributed (Present: p = .06; Rest: p = .10; Shapiro-Wilk normality test), but within-Pain and across-network correlation measures were not (Present: ps<.0002; Rest: ps<.00005).

In order to test for developmental change in within- and across-network correlations, linear regressions were used to test for 1) significant effects of age (as a continuous variable) in the full sample (ages 5–20 years), in regressions that additionally included number of artifact timepoints as a predictor, and 2) significant effects of age (as a continuous variable), SCQ, and number of artifact timepoints among children. In order to test whether ToM and pain networks are functionally dissociated early in childhood, t-tests were conducted to compare within- versus across-network correlations in five-year-old children (n = 16).

#### 2.4.2 Reverse correlation analyses

Initial reverse correlation analyses of “The Present” task were conducted on adolescent/young adult participants only (n = 55), for identification of events. Each ROI timecourse was z-normalized, and timecourses within each network were averaged across ROIs, resulting in one timecourse for the ToM network and one timecourse for the Pain Matrix per participant. Except for the first five timepoints (4 s), the residual signal values across adult subjects for each timepoint were tested against baseline (0) using a one-tailed *t*-test. Events were defined as five or more consecutive significantly positive timepoints within each network (i.e., as in the previous study (Richardson et al., 2018), events were at least 4 s in duration). Because reverse correlation analyses compare responses across participants to baseline, identified events should be considered to drive responses in the regions of interest relative to other moments in the stimulus. Events were rank-ordered according to the average magnitude of response to the peak timepoint, and labeled according to the ordering (i.e. event T01 is the ToM event that evoked the highest magnitude of response in the ToM network).

In order to test for developmental effects in the magnitude of response to ToM and pain events, a peak timepoint was defined for each event as the timepoint with the highest average signal value in adults, and the correlation between the magnitude of response at peak timepoints and age (as a continuous variable), including amount of motion (number of artifact timepoints) as a covariate, was measured. Response magnitude to eight of ten events was normally distributed (all ps>.06, Shapiro-Wilk normality test); response magnitude to events T03 and P02 were non-normally distributed among children (ps<.02). Because the number of artifact timepoints is a non-normally distributed measure, spearman correlation tests were used for all events. For ToM regions only, linear regressions were used to test for a significant relationship between peak magnitude of response and score on the Social Communication Questionnaire (SCQ). A Bonferroni correction was used to correct for multiple comparisons (age: 10 events tested; SCQ: 7 events tested). As in the previous study (Richardson et al., 2018), the reverse correlation analysis was also conducted in the youngest children scanned (five-year-old participants; n = 16).

#### 2.4.3 Functional maturity

Finally, the correlation between the functional maturity of each child’s timecourse responses (i.e. similarity to adolescents/young adults) during “The Present” and inter-region network correlations was measured. The Pearson correlation between each child’s ToM timecourse (averaged across ROIs) and the average adult ToM timecourse was calculated; the same procedure was used to calculate functional maturity in the Pain Matrix. The timecourses used for this analysis were the same as those used for the reverse correlation analysis, prior to z-normalization (TRs 6:250). Correlation tests were conducted to test if, across children, this measure of functional maturity per network was correlated with within-network and across-network inter-region correlations, or to SCQ score. The functional maturity measure was normally distributed in the Pain (p = .10, Shapiro-Wilk normality test) but not ToM network (p = .004). The Pearson correlation between the average timecourse of children (all children, and five year olds separately) and the average adolescent/young adult timecourse was also measured.

Linear regressions were used to test for correlations between functional maturity and inter-region correlations as measured during movie-viewing and during rest. These regressions controlled for age (continuous variable) and motion.

### 2.5 Comparison to previous results

For easy comparison to the prior study, the data from that study were reanalyzed in 5–12 year old children only (i.e., excluding 3–4 year olds). The analysis procedures were identical to those described above; the participants and experimental paradigm were described previously (Richardson et al., 2018). The results of these analyses are included in the Supplementary Materials.

### 2.6 Behavioral measures

The Social Communication (SCQ (Rutter et al., 2003)) score was used to measure individual differences in social cognition. The SCQ is a parent questionnaire that is often used to screen individuals for Autism Spectrum Disorder. Items tap a wide array of social behaviors, including aspects of conversation, interests, physical behaviors (e.g., flapping fingers, but also pointing/gesturing), sharing, helping, and empathic responses. Two phenotypic measurements collected by the Child Mind Institute that characterize social behavior were initially downloaded: the SCQ and the Social Responsiveness Scale (SRS (Constantino and Gruber, 2012)). While the Child Mind Institute is additionally conducting ADOS screening (Lord et al., 2000), these data are not yet publicly available. The SRS and SCQ measures were significantly positively correlated in the current sample (*r*_s_(191) = .70, p < 2.2 × 10^−16^), even when including age as a covariate (p < 2 × 10^16^). Neither of these measures were normally distributed (Shapiro-Wilk normality test: ps<3.4 × 10^-7^). Because these measures were highly correlated, and the SCQ task is identical for all participants (whereas younger participants complete a different version of the SRS), SCQ scores were used as the behavioral measure of individual differences in social cognition.

## 3 Results

This study first sought to confirm results of a previous exploratory study on children (n = 122, 3–12 years) and adults (n = 33) who watched Pixar’s animated short “Partly Cloudy” while undergoing fMRI. The previous study found that 1) ToM and pain networks are functionally dissociated by age three years, 2) network differentiation increases throughout childhood, 3) the magnitude of response during one scene is correlated with cognitive performance on a test of ToM, and 4) the “functional maturity” of the response timecourse is correlated with the inter-region correlations of the networks. The current study tested whether these results replicate in an independent, large, and heterogeneous sample of participants who viewed a different movie (Jacob Frey’s “The Present” (2014)) while undergoing fMRI.

### 3.1 Replication: inter-region correlation analyses

**Fig. 1:**
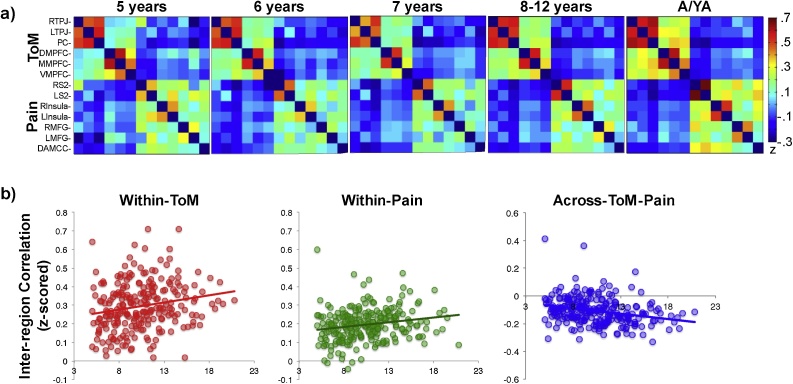
**Developmental Change in Inter-region Correlations. a)** Average z-scored correlation matrices across all ToM and pain brain regions of interest (see y-axis) per age group (5yo: n = 16; 6yo: n = 20; 7yo: n = 26; 8-12yo: n = 123; adolescents/young adults (YA; 13–20 years): n = 53), as measured during movie-viewing. Regions are in the same order along the X-axes and Y-axes. **b)** Z-scored inter-region correlations (y-axis) by age (x-axis) within the ToM network (left, red), within the Pain network (middle, green), and across the ToM-Pain networks (right, blue). (For interpretation of the references to colour in this figure legend, the reader is referred to the web version of this article).

As in the original study, ToM and Pain brain regions were significantly more correlated with within-network brain regions, compared to brain regions in the opposite network. A significantly positive within – across network correlation difference is one indication of a functional dissociation. Among adolescents and young adults (A/YA), within-network correlations (M(SE) Wi-ToM: .34(.02), Wi-Pain: .23(.01)) were significantly higher than across-network correlations in both networks (M(SE) ac-TP: -.15(.01); within vs. across-network two-tailed paired *t-tests*: **ToM:** t(52) = 21.4, p < 2.2 × 10^−16^; **Pain:** t(52) = 22.9, p < 2.2 × 10^−16^). Within-network correlations were significantly positive (ps<2.2 × 10^−16^) and the across-network correlation was significantly negative (t(52)=−12.8, p < 2.2 × 10^−16^); see Fig. 1.

The current study also tested for developmental change in inter-region correlations. Because the age range of the current sample (ages 5–20 years) differs from that of the previous study (ages 3–12 years, and adults), primary inter-region correlation analyses were conducted in the full sample, and results from the age-matched child sample (5–12 year olds) are additionally reported. See Supplementary Materials for the results of age-matched analyses in the prior study. Consistent with the previous results, within-network inter-region correlations increased significantly with age and across network inter-region correlations decreased significantly with age (full sample: ps<.005); see Fig. 1 and Supplementary Fig. 3 for visualizations, see Table 1 for statistics. Within-network age effects become marginal in the low/matched motion sample (ps<.1); the across-network age effect remains significant (p = .02). In both studies, developmental change with age was less apparent in the narrower, matched age range (5–12 year old children): there was not significant evidence for developmental change in 5–12 year old children in within- or across-network correlations, but all correlations showed developmental trends in the predicted directions (Table 1 and Supplementary materials). Age correlations among 13–20 year old participants were non-significant (ps > .15).

**Table 1:** Inter-region Correlation Analyses: Developmental Change with Age.

IRC measured during "The Present"	Predictor	Full Sample (n = 238, 5-20 years)	Child Sample (n = 185, 5-12 years)	Full Matched Sample (n = 106, 5-19 years)	Child Matched Sample (n = 81, 5-12 years)
Within-ToM	Age	**b = .15, t** **=** **2.4, p =** **.02**	b = .14, t = 1.9, p = .055	b = .17, t = 1.7, p = .09	b = .12, t = 1.1, p = .29
	Motion	**b = −.22, t = −3.4, p = .0007**	**b = −.22, t = -3.1, p = .002**	b = −.15, t = −1.5, p = .13	b = −.19, t = −1.7, p = .09
Within-Pain	Age	**b = .17, t = 2.6, p=.009**	b = .11, t = 1.5, p = .12	b = .19, t = 1.9, p = .06	b = .15, t = 1.4, p = .18
	Motion	b = −.12, t = −1.8, p = .08	b = −.13, t = −1.8, p = .07	b = −.08, t = −.79, p = .43	b = −.09, t = −.77, p = .44
Across-ToM-Pain	Age	**b = −.20, t = −3.2, p=.001**	b = −.12, t = −1.7, p = .10	**b = −.24, t = −2.4, p = .02**	b = −.20, t = −1.8, p = .08
	Motion	**b = .31, t** = **5.0, p = 9.9 × 10**^**−7**^	**b = .33, t = 4.7, p = 4.7 × 10**^**−6**^	b = −.02, t = −.24, p = .82	b = −.09, t = −.78, p = .44

IRC measured At Rest	Predictor	Full Sample (n = 200, 5-20 years)	Child Sample (n = 151, 5-12 years)	Full Matched Sample (n = 106, 5-19 years)	Child Matched Sample (n = 81, 5-12 years)
Within-ToM	Age	**b =** **.37, t=5.6, p=6.7 × 10**^**−8**^	b = .14, t = 1.8, p = .07	**b = .25, t =** **2.8, p=.006**	b = .09, t = .80, p = .42
	Motion	**b = −.20, t = −3.1, p =** **.002**	**b = −.42, t = −5.7, p = 7.3 × 10**^**−8**^	**b = −.32, t = −3.5, p = .0008**	**b = −.37, t = −3.5, p = .0007**
Within-Pain	Age	**b =** **.26, t = 3.7, p =** **.0003**	b = .07, t = .83, p = .41	**b = .22, t = 2.3, p = .03**	b = .07, t = .63, p = .53
	Motion	b = −.002, t = −.03, p = .97	b = −.03, t = −.40, p = .69	**b = −.24, t = −2.5, p = .01**	**b = −.24, t = −2.12, p = .04**
Across-ToM-Pain	Age	**b = −.45, t = −6.9, p =** **5.4 × 10**^**−11**^	**b = −.29, t = −3.6, p = .0005**	**b = −.38, t = −4.3, p = 3.7 × 10**^**−5**^	b = −.13, t = −1.2, p = .23
	Motion	b = .04, t = .60, p = .55	b = .18, t = 2.3, p = .03	**b = .26, t** **=** **2.9, p =** **.004**	**b = .29, t = 2.7, p = .009**

**Statistics for Regressions Testing for Developmental Change in Inter-region Correlations.** Statistics include standardized beta values, t-statistics, and p-values for each predictor included in each regression. Age is a continuous variable in all regressions. Matched Samples include participants with low- and matched-amounts of motion in the movie and resting state scans; child samples include 5–12 year old participants. Results that are significant at p < .05 are in bold font.

The previous study found evidence for functionally dissociated ToM and Pain networks in children as young as three years old. In the current sample, the youngest children scanned were five years old. In these children, within-network correlations were significantly greater than across-network correlations (n = 16 5yo; M(SE) within-ToM: .25(.04), within-Pain: .19(.03), across-ToM-Pain: -.07(.04); within vs. across-network correlation paired two-tailed *t*-test: **ToM:** t(15) = 7.3, p = 2.7 × 10^−6^, **Pain:** t(15) = 7.0, p = 4.5 × 10^−6^). Given the small sample size, these results were confirmed with non-parametric Wilcoxon signed rank tests (Vs = 136, ps<.00005). As with the adolescents and young adults, within-network correlations were significantly positive (ps<.00005). However, the across-network correlation did not differ significantly from zero (t(15)=−1.7, p = .10).

The previous study found a significant relationship between behavioral performance on a ToM behavioral battery and within-ToM and across-ToM-Pain inter-region correlations, but these relationships did not remain significant when additionally controlling for age. The current study used scores on the Social Communication Questionnaire (SCQ (Rutter et al., 2003)), a parent report questionnaire, as a measure of social cognitive reasoning. Scores on the Social Communication Questionnaire significantly correlated with age in the full sample (*r_s_*(193) = .19, p = .01), reflecting more variable and high scores among older participants who contributed fMRI data; this correlation was marginal in the child sample (*r_s_*(151) = .16, p = .05). There were no significant correlations between within-ToM or across-ToM-Pain inter-region correlations and SCQ scores among children (partial correlations including motion as covariate: *r*s<|.11|, ps>.2), or in the full sample (*r*s<|.06|, ps>.4).

### 3.2 Replication: reverse correlation analyses

**Fig. 2:**
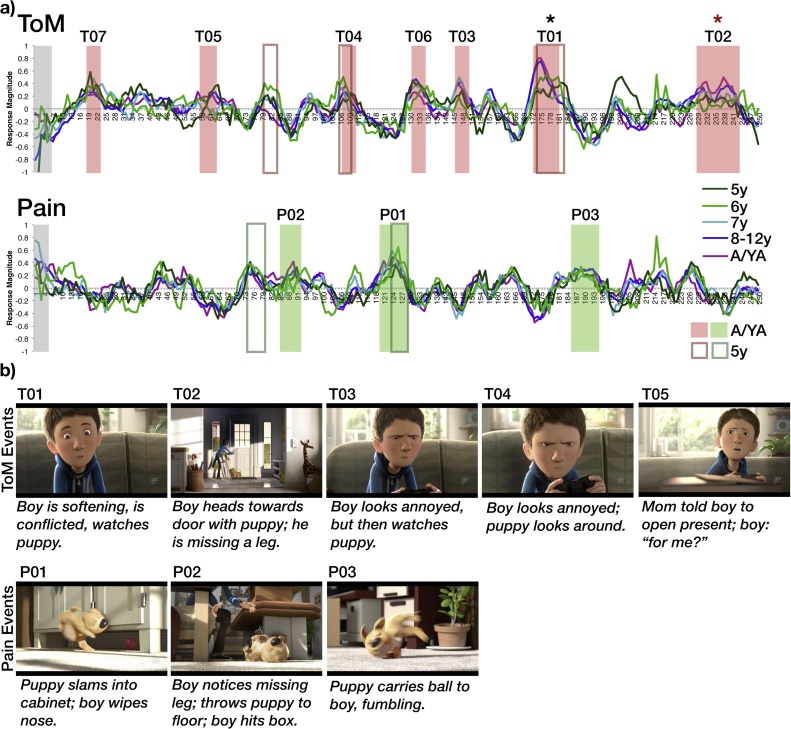
**Functional Timecourses during “The Present.” a)** The average timecourse per age group for the ToM network (top) and Pain matrix (bottom), during viewing of Jacob Frey’s “The Present” (2014). Each timepoint along the x-axis corresponds to a single TR (800 ms); the entire movie was 250 TRs (<4 min). Shaded blocks show timepoints identified as ToM (red) and Pain (green) events in a reverse correlation analysis conducted on adolescent/young adult participants (13–20 year olds; n = 55); timepoints within the gray block were not analyzed. Dark red and green borders show timepoints identified as ToM and pain events, respectively, in 5-year-old children (n = 16). Event labels (e.g., T01, P01) indicate ranking of average peak magnitude of response in adolescents/young adults. Black asterisk indicates significant positive correlation between peak magnitude of response and age (continuous variable) among children, after correcting for multiple comparisons (10 ToM/Pain events, **α** = .005). Red asterisk indicates significant positive correlation between peak magnitude of response and SCQ score (continuous variable) among children; this correlation does not survive correcting for multiple comparisons (7 ToM events; **α** = .007, p = .02). **b)** Example frames and descriptions for the five events with the highest magnitude of response in adolescents/young adults, per network (see Supplementary Fig. 5 for all events, and Supplementary Table 1 for full event descriptions and timing and duration information). Thumbnail images used with permission from Jacob Frey (For interpretation of the references to colour in this figure legend, the reader is referred to the web version of this article).

Reverse correlation analyses offer a data-driven way to determine what kinds of stimuli drive responses in particular brain regions. A reverse correlation analysis was conducted on the neural responses in adolescent and young adult participants (n = 55) while they watched “The Present” (Frey, 2014). This analysis produced seven ToM events (40 s total, M(SD) length: 5.7(3.0) seconds) and three Pain events (21.6 s total, M(SD) length: 7.2(1.4) seconds); see Fig. 2. Six of the seven ToM events clearly depicted moments that involved reasoning about mental states (beliefs, goals, emotions) of the characters (e.g., boy curiously opening present, boy expressing annoyance, and gaining a new understanding of the boy, upon realizing that he, like the puppy, has lost his leg). The remaining ToM event introduced the boy character and showed him playing video games. The three Pain events depicted moments involving physical pain or bodily clumsiness (due to the missing leg). See Supplementary Table 1 for more information about the timing and content of the events. Note that while these events are referred to as “Pain events” in order to refer to the Pain Matrix ROIs used for analysis, the content that evokes responses in these regions is not limited to physical pain, but also includes moments that focus on bodily movements and sensations (as in prior studies (Lombardo et al., 2010; Spunt et al., 2015)). Out of the 245 timepoints tested (all but the first 5 TRs (4 s)), there were zero timepoints that reliably evoked significantly positive responses in both ToM and Pain events.

Responses to one ToM event (T01) increased significantly with age among 5–12 year old children (partial spearman correlation test, including motion as a covariate: *r*_s_(175) = .23, p = .0024; Bonferroni correction for multiple comparisons **α** = .005, for ten events; event P02: *r*_s_(175) = .19, p = .009; all other events: ps>.02). Responses to a second ToM event (T02) positively correlated with SCQ score in a linear regression that included age and motion as covariates (effect of SCQ: -.21, t=-2.4, p = .018, effect of age: b = .19, t = 2.3, p = .02, NS effect of motion: b=-.05, t=-.57, p = .57; all other events SCQ ps>.12); this relationship did not survive Bonferroni correction for multiple comparisons (**α** = .007, for seven ToM events).

As in the previous study, a reverse correlation analysis was also conducted on the youngest participants scanned (age 5 years old, n = 16). Responses in five year olds were generally highly correlated with the average adolescent/young adult timecourse (M(SE) of Pearson correlation: ToM: .23(.04), Pain: .22(.04); one sample t-tests against zero: ts(15) = 6.1, ps<2.2 × 10^−5^). In five year olds, the reverse correlation analysis identified two of the seven ToM events and one of the three Pain events defined in the adolescent/young adult participants; see Fig. 2. These events made up a majority of the timepoints identified as events in the five year olds (18/32 TRs). Three of the remaining 14 TRs immediately preceded or followed these events. The remaining 11 TRs comprised one ToM event and one Pain event, which shared a single timepoint (6 TRs each); neither of these events were identified in the adolescent/young adult sample. See Supplementary Figure 5 and Supplementary Table 1 for more information about all events.

### 3.3 Replication: functional maturity

The current dataset included adolescents/young adults (13–20 years old), rather than adults (in the previous study: ages 18–39 years old). Response timecourses among 5–12 year old children were generally positively correlated with the average timecourse of adolescents and young adults (n = 186 5-12yo: M(SE) Pearson correlation value (*r*): ToM: .30(.01), Pain: .27(.01)). However, as in the previous study, functional maturity (i.e., similarity to responses in adolescents/young adults) in ToM and Pain networks increased with age among 5–12 year old children (spearman partial correlations including motion as a covariate: ToM: *r*_s_(182) = .20, p = .006; Pain: *r*_s_(182) = .19, p = .01). Functional maturity in the ToM network was not significantly correlated with SCQ score (spearman partial correlation including motion as a covariate: *r*_s_(150) = .08, p = .35).

**Fig. 3:**
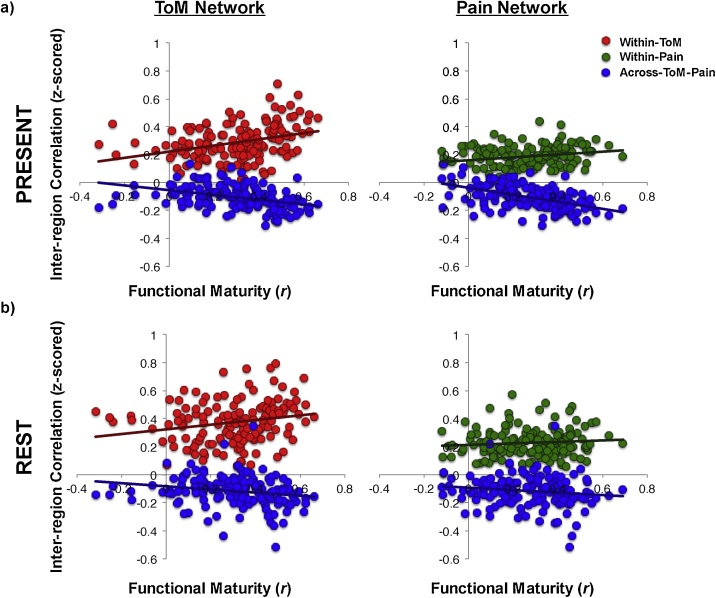
**Relating Functional Maturity to Inter-region Correlations.** Scatterplots show timecourse maturity (i.e., how correlated each child’s timecourse is to the average adolescent/young adult timecourse (Pearson’s *r*, x-axis) while viewing Jacob Frey’s “The Present” (2014). The y-axis shows z-scored inter-region correlation values within-ToM (red), within-Pain (green), and across-ToM-Pain (blue) networks, as measured while viewing **a)** “The Present”, or **b)** at rest. (For interpretation of the references to colour in this figure legend, the reader is referred to the web version of this article).

There were significant effects of within- and across-network correlations on functional maturity in the ToM network; see Fig. 3a for visualization and Table 2 for statistics. In the Pain network, only the across-network correlation significantly predicted functional maturity (Fig. 3a, Table 2). This same pattern of results was apparent in a low/matched motion subset of participants who contributed fMRI data to the movie and resting state scans (n = 106; including n = 81 5-12yo). In this subset, functional maturity in both networks was predicted by the anti-correlation between the two networks (Table 2). VIF scores for all predictors were <2 in these regressions.

**Table 2:** Inter-region Correlation Analyses: Relationship to Functional Maturity.

IRC measured during "The Present"	Predictor	Child Full Sample (n = 185)	Child Matched Sample (n = 81)
Functional Maturity in ToM Network	Across-ToM-Pain IRC (Movie)	**b = −.17, t = −2.2, p = .03**	**b = −.39, t = −2.8, p** = **.007**
	Within-ToM IRC (Movie)	**b** = **.25, t** = **3.4, p** = **.0008**	b = .10, t = .75, p = .46
	Age	b = .13, t = 1.8, p = .07	b = .03, t = .28, p = .78
	Motion	b = −.09, t = −1.3, p = .21	b = −.20, t = −1.9, p = .07
Functional Maturity in Pain Network	Across-ToM-Pain IRC (Movie)	**b = −.41, t = −5.9, p** = **1.5 × 10**^**−8**^	**b = −.43, t = −3.9, p** = **.0002**
	Within-Pain IRC (Movie)	b = .11, t = 1.6, p = .11	b = .08, t = .74, p = .46
	Age	b = .13, t = 1.9, p = .06	b = .01, t = .93, p = .36
	Motion	b = −.07, t = −1.0, p = .30	b = −.05, t = −.50, p = .62

IRC measured at Rest	Predictor	Child Full Sample (n = 151)	Child Matched Sample (n = 81)
Functional Maturity in ToM Network	Across-ToM-Pain IRC (Rest)	b = −.07, t = −.66, p = .51	b = −.12, t = −.84, p = .40
	Within-ToM IRC (Rest)	b = .10, t = .92, p = .36	b = −.06, t = −.41, p = .68
	Age	b = .15, t = 1.7, p = .10	b = .07, t = .66, p = .51
	Motion	b = −.13, t = −1.4, p = .16	**b = −.25, t = −2.1, p** = **.04**
Functional Maturity in Pain Network	Across-ToM-Pain IRC (Rest)	b = −.03, t = −.35, p = .73	b = −.06, t = −.46, p = .64
	Within-Pain IRC (Rest)	b = .07, t = .80, p = .43	b = .11, t = .81, p = .42
	Age	b = .12, t = 1.4, p = .16	b = .18, t = 1.6, p = .12
	Motion	**b = −.18, t = −2.2, p** = **.03**	b = −.06, t = −.52, p = .61

**Statistics for Regressions testing for Correlations between Inter-region Correlations and Functional Maturity.** Statistics include standardized beta values, t-statistics, and p-values for each predictor included in each regression. Age is a continuous variable in all regressions. Child Matched Sample includes participants with low- and matched-amounts of motion in the movie and resting state scans; both child samples include 5–12 year old participants. Results that are significant at p < .05 are in bold font.

### 3.4 Extension: inter-region correlations during resting state

A subset of the sample completed a resting state scan (n = 200), enabling this study to test if the pattern of results from the inter-region correlation analyses were specific to functional responses during a social, naturalistic movie-viewing paradigm. Within-network correlations (M(SE) within-ToM: .51(.02), within-Pain: .29(.02)) were higher than across-network correlations (M(SE) across-ToM-Pain:-.23(.02)) during rest in adolescents/young adults (within vs. across-network correlation paired two-tailed *t*-test: **ToM:** t(48) = 21, p < 2.2 × 10^−16^; **Pain:** t(48) = 19.2, p < 2.2 × 10^−16^).

**Fig. 4:**
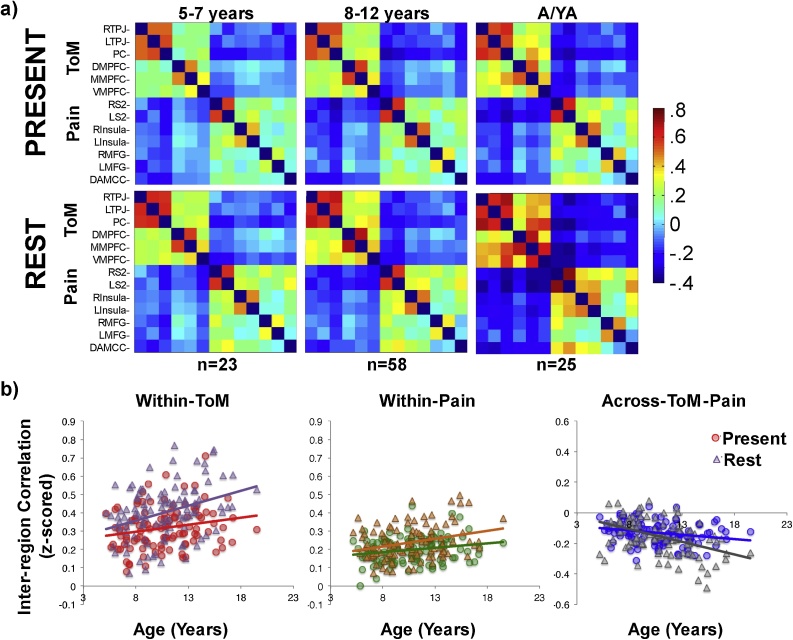
**Inter-region Correlations during Movie-Viewing and at Rest. a)** Average z-scored correlation matrices across all ToM and pain brain regions of interest (see y-axis) in low/matched motion participants, as measured while viewing Jacob Frey’s “The Present” (2014) (top row), or at rest (bottom row), by age group (5–7 years: n = 23; 8–12 years; n = 58; adolescents/young adults (A/YA): n = 25). **b)** Z-scored inter-region correlations (y-axis) by age (x-axis) within the ToM network (left, red/purple), within the Pain network (middle, green/orange), and across the ToM-Pain networks (right, blue/grey). Circles show inter-region correlations as measured during Jacob Frey’s “The Present” (2014); triangles show inter-region correlations as measured during rest. (For interpretation of the references to colour in this figure legend, the reader is referred to the web version of this article).

In the full sample (ages 5–20 years), within-network inter-region correlations during rest increased significantly with age (ps<.0005), and across-network inter-region correlations decreased significantly with age (p = 5.4 × 10^−1^) see Table 1 and Fig. 4. An analysis of resting state data in the low/matched motion subset of participants (n = 106) yielded the same pattern of results. Among 5–12 year old children, within-ToM and within-Pain network correlations did not increase significantly with age in the full sample or in the low/motion-matched sample, consistent with the results from the movie-viewing task (Table 1). Across-network correlations during rest decreased with age in the full sample of 5–12 year old children (p = .0005); this result was not significant in the low/motion-matched 5–12 year old sample (p = .23; Table 1). Age effects among 13–20 year old participants were not significant (ps>.15). There were no significant correlations between within-ToM or across-ToM-Pain inter-region correlations measured at rest and SCQ scores among children (partial correlations including motion as covariate: *r*s<|.08|, ps>.4), or in the full sample (*r*s<|.04|, ps>.6).

Very few five year olds were included in the resting state sample (n = 7). However, even in this small sample, within-network correlations (M(SE) within-ToM: .32(.03), within-Pain: .26(.04)) were significantly higher than across network correlations (M(SE): -.08(.08); within vs. across-network correlation paired two-tailed *t*-test: **ToM:** t(6) = 4.0, p = .008; **Pain:** t(6) = 4.0, p = .007). Given the small sample, these results were confirmed with non-parametric Wilcoxon signed rank tests (**ToM:** V = 27, p = .03, **Pain:** V = 28, p = .02). As in the movie-viewing task, within-network correlations were significantly positive (ps<.001), and across-network correlations did not differ from zero (t(6)=-.99, p = .36).

### 3.5 Extension: inter-region correlations during resting state vs. movie-viewing

In the low/matched-motion sample, all inter-region correlation measures were highly correlated across movie-viewing and rest scans, even when controlling for age and motion (**Rest IRCs as predictors of movie-viewing IRCs:** within-ToM: b = .50, t = 5.2, p = 1.04 × 10^−6^, age and motion effects: ps>.7; within-Pain: b = .49, t = 5.2, p = 9.3 × 10^-7^, age and motion effects: ps>.4; across-ToM-Pain: b = .47, t = 4.5, p = 1.7 × 10^-5^, age and motion effects: ps>.6; **Movie-Viewing IRCs as predictors of Rest IRCs:** within-ToM: b = .43, t = 5.2, p = 1.04 × 10^−6^, age: b = .18, t = 2.2, p = .03, motion: b=-.24, t=-2.9, p = .004; within-Pain: b = .43, t = 5.2, p = 9.3 × 10^-7^, age: b = .13, t = 1.5, p = .15, motion: b=-.23, t=-2.7, p = .008; across-ToM-Pain: b = .36, t = 4.5, p = 1.7 × 10^-5^, age: b=-.30, t=-3.6, p = .0005, motion: b = .25, t = 3.0, p = .003; Note: all age x IRC interactions were non-significant and not included in these regressions); see Supplementary Fig. 4.

The collection of resting state data in addition to movie-viewing data enabled testing if the developmental separation of functional responses (i.e. within – across network correlation difference) in the ToM and pain networks differed by task (“The Present” vs. resting state). This analysis was conducted on a subset of participants (n = 106; including n = 81 5-12yo) who had low and matched amount of motion in these scans. Mixed effect regressions were used to test for main effects of age, task (movie vs. rest), motion, and a task-by-age interaction, on the within – across network correlation difference, per network. If the task-by-age interaction was non-significant, the regression was repeated without the interaction term and statistical evidence was reported from this second regression. Regressions included a subject identifier as a random effect in order to account for non-independence of data across the two tasks.

In the full sample (n = 106), the within – across network correlation difference in both networks was larger during the resting state scan, and there was a significant task-by-age interaction such that the positive effect of age on the within – across difference was stronger as measured during rest (effects of task: ps<.0005, task-by-age interactions: ps<.01, see Table 3). Among 5–12 year old children (n = 81), the within – across network difference in both networks did not increase significantly with age, and was larger as measured during rest (effects of task: ps<.05, Table 3); see Fig. 4. The within – across network difference was similarly not significantly correlated with age among 13–20 year old participants (ps > .15).

**Table 3:** Inter-region Correlation Analyses: Developmental Change with Age by Task.

IRC Measure	Predictor	Full Matched Sample (n = 106, 5-19 years)	Child Matched Sample (n = 81, 5-12 years)
Within-Across ToM	Task (Rest > Movie)	**b** = **.50, t** = **5.5, p** = **2.8 × 10**^**−7**^	**b** = **.39, t = 3.6, p** = **.0006**
	Age	b = .15, t = 1.8, p = .08	b = .15, t = 1.6, p = .11
	Motion	**b = −.19, t = −2.9, p** = **.005**	**b = −.21, t = −2.7, p** = **.008**
	Task x Age	**b** = **.26, t** = **2.9, p = .005**	*NS; not included in final regression*
Within-Across Pain	Task (Rest > Movie)	**b** = **.39, t** = **4.1, p** = **.0001**	**b** = **.26, t** = **2.3, p** = **.03**
	Age	b = .17, t = 1.9, p = .06	b = .17, t = 1.8, p = .07
	Motion	**b = −.15, t = −2.2, p** = **.03**	**b = −.16, t = −2.0, p** = **.049**
	Task x Age	**b** = **.27, t = 2.8, p** = **.006**	*NS; not included in final regression*

IRC Measure	Predictor	Full Matched Sample (n = 106, 5-19 years)	Child Matched Sample (n = 81, 5-12 years)
Within-ToM	Task (Rest > Movie)	**b** = **.62, t** = **6.7, p** = **8.7 × 10**^**−10**^	**b = .57, t** = **5.3, p** = **1.04 × 10**^**−6**^
	Age	**b** = **.21, t** = **2.8, p** = **.006**	b = .10, t = 1.2, p = .23
	Motion	**b = −.22, t = −3.4, p** = **.001**	**b = −.25, t = −3.3, p** = **.002**
	Task x Age	*NS; not included in final regression*	*NS; not included in final regression*
Within-Pain	Task (Rest > Movie)	**b** = **.49, t = 5.1, p** = **1.9 × 10**^**−6**^	**b** = **.44, t** = **3.8, p** = **.0003**
	Age	**b** = **.20, t** = **2.6, p** = **.01**	b = .11, t = 1.2, p = .23
	Motion	**b = −.15, t = −2.2, p** = **.03**	b = −.16, t = −2.0, p = .052
	Task x Age	*NS; not included in final regression*	*NS; not included in final regression*
Across-ToM-Pain	Task (Rest > Movie)	**b = −.22, t = −2.2, p** = **.03**	b = −.03, t = −.24, p = .81
	Age	b = −.15, t = −1.7, p = .09	b = −.17, t = −1.9, p = .07
	Motion	b = .13, t = 1.9, p = .07	b = .12, t = 1.5, p = .14
	Task x Age	**b = −.34, t = −3.4, p** = **.001**	*NS; not included in final regression*

**Statistics for Regressions testing for Age and Task Effects on Inter-region Correlations.** Statistics include standardized beta values, t-statistics, and p-values for each predictor included in each regression. Age is a continuous variable in all regressions. Full and Child Matched Samples were participants with low- and matched-amounts of motion in the movie and resting state scans. Results that are significant at p < .05 are in bold font.

Follow-up analyses were conducted to determine which aspect of the within – across network difference drove the developmental increase in network dissociation at rest. In the motion-matched sample, there was a significant task-by-age interaction on the across-network correlation, such that the across-ToM-Pain network correlation decreased more with age as measured at rest (p = .001, see Table 3). There were not significant task-by-age interactions on the within-ToM and within-Pain network correlations (ToM: p = .07; Pain: p = .21, interaction terms not included in final regressions). There were no significant task-by-age interactions on the across-ToM-Pain, within-ToM, or within-Pain network correlations among the 5–12 year old subset (ps > .7).

Finally, linear regressions were conducted to test if inter-region correlations measured at rest were correlated with the “functional maturity” of the response (as measured during movie-viewing), among children (5–12 years old). In both networks, neither within-network or across-network inter-region correlations were significantly correlated with functional maturity (ps>.3). The same pattern of evidence was apparent in the low/matched-motion subset of participants; see Fig. 3b for visualization and Table 2 for statistics. All age-by-inter-region correlation interaction terms were non-significant (ps>.24) and not included in these regressions, and VIF scores for all predictors in these regressions were <2.

Linear regressions in the low-motion subset of participants (n = 106) were used to simultaneously test for effects of inter-region correlations as measured at rest and as measured during movie-viewing on functional maturity, per network. The predictors included in these regressions were: across-TP-movie, across-TP-rest, within-[ToM or Pain]-movie, within-[ToM or Pain]-rest, age, motion (average number of artifact timepoints across the movie and resting state scans). In the ToM network, functional maturity was predicted by the across-ToM-Pain correlation as measured during movie-viewing (p = .005; all VIF scores < 2.2). In the Pain Matrix, functional maturity was predicted by the across-ToM-Pain correlation as measured during the movie and at rest (effect of across-TP-movie: b=-.54, t=-4.7, p = 1.2 × 10^−5^, effect of across-TP-rest: b = .33, t = 2.5, p = .02; all VIF scores < 2); see Table 4 for full statistics. Although the VIF scores for these regressions were low, the sign flip on the across-TP-rest predictor for the Pain Matrix regression and the within-ToM-rest predictor for the ToM regression may reflect the multi-collinearity of the predictors (Yoo et al., 2014). Given this, this pattern of results was confirmed using lasso regressions; see Supplementary Materials.

**Table 4:** Simultaneous Test of Effects of IRCs Measured at Rest and During "The Present" on Functional Maturity in ToM and Pain Networks.

Network	Predictor	Child Matched Sample (n = 81)
Functional Maturity in ToM Network	Across-ToM-Pain IRC (Movie)	**b = -.40, t = -2.9, p = .005**
	Across-ToM-Pain IRC (Rest)	b = .04, t = .32, p = .75
	Within-ToM IRC (Movie)	b = .20, t = 1.4, p = .16
	Within-ToM IRC (Rest)	b = -.22, t = -1.5, p = .13
	Age	b = .02, t = .19, p = .85
	Motion	**b = -.31, t = -2.8, p = .006**
Functional Maturity in Pain Network	Across-ToM-Pain IRC (Movie)	**b = -.54, t = -4.7, p = 1.2 × 10^−5^**
	Across-ToM-Pain IRC (Rest)	**b = .33, t = 2.5, p = .02**
	Within-ToM IRC (Movie)	b = .09, t = .83, p = .41
	Within-ToM IRC (Rest)	b = .09, t = .73, p = .47
	Age	b = .11, t = 1.0, p = .30
	Motion	b = −.13, t = −1.2, p = .23

**Statistics for Regression testing for Correlations between Inter-region Correlations and Functional Maturity.** Statistics include standardized beta values, t-statistics, and p-values for each predictor included in each regression. Age is a continuous variable in all regressions. The Child Matched Sample includes 5–12 year old participants with low- and matched-amounts of motion in the movie and resting state scans. Results that are significant at p < .05 are in bold font.

Prompted by visualizing the data (Fig. 3), additional exploratory analyses found greater variance in inter-region correlation measures during rest, relative to during movie-viewing (F tests comparing wi-ToM measured during movie/at rest: F(105) = .61, p = .01; wi-Pain: F(105) = .60, p = .01; across-TP: F(105) = .43, p = 2.6 × 10^−5^; 5–12 year olds only: wi-ToM: F(80) = .65, p = .06; wi-Pain: F(80) = .69, p = .09; across-TP: F(80) = .56, p = .01).

## 4 Discussion

One challenge in developmental cognitive neuroscience, developmental psychology, and cognitive neuroscience is to design and execute studies that are easily replicable (Munafò et al., 2017) as well as generalizable to diverse samples (Falk et al., 2013). “Big Data” offers one way to address this challenge, by providing large datasets that enable discovery and replication of patterns or principles of brain development. The current study involved analyzing a large, publicly available fMRI dataset (Alexander et al., 2017) in order to replicate and extend the results of a previous exploratory fMRI study. A key goal was to determine the robustness of previously identified neural markers of brain development that relate to behavioral measures of social cognition.

This study provides confirmatory evidence that two networks involved in social responding, the ToM and pain networks, are functionally dissociated in children as young as five years of age. Inter-region correlation analyses revealed strong, positive correlations between brain regions within each network, and anti-correlated responses across the two networks. Reverse correlation analyses identified distinct events that evoked responses in each network; these events were consistent with previous evidence that ToM brain regions preferentially respond to scenes that highlight mental states (beliefs, desires, emotions), and the “Pain Matrix” preferentially responds to scenes that highlight bodily sensations (e.g., physical pain, bodily movements).

While responses in both networks in children were generally highly correlated with the average timecourse of responses in adolescents and young adults, there was significant developmental change in functional responses to the movie. Responses to one ToM event (T01) increased significantly with age. This event showed the boy, who had previously expressed annoyance at the three-legged puppy his mother gave him, softening, and feeling conflicted about softening, while watching the puppy. As in the previous study, the event that showed change with age was a relatively long event, requiring complicated mental state reasoning, and was the event with the highest response magnitude in adults.

One benefit of conducting confirmatory analyses in publicly available datasets is that it tests the generalizability of results to samples that are more heterogeneous than those typically acquired by a single lab. Indeed, the current sample had a large range of SCQ scores, and included participants whose scores are above typical cut-offs indicating social difficulties. Given the range and variability of SCQ scores, this dataset could offer a more sensitive test case for how real world variability relates to variability in neural responses. In fact, just like the in previous study, the magnitude of response to a particular ToM event (T02) correlated with SCQ score. While this result does not survive correcting for multiple comparisons across all seven ToM events, event T02 bears the most resemblance to the kind of event that was related to ToM behavior in the prior study (Richardson et al., 2018). Event T02 involves the revelation (for the audience members) that the boy, too, is missing a leg. In the context of the movie, this scene provides insight into the boy’s behavior: he was initially put off by the puppy’s missing leg because he is adapting to his own physical limitations, but eventually warms up to the puppy and feels encouraged to play outside rather than sit inside and play video games all day. As in the previous study, increased activity in ToM regions during this event may reflect children’s improved ability to spontaneously consider the relevance of the current scene for past beliefs or emotions that are not explicitly marked. Together, these results suggest that measures of spontaneous mental state reasoning may be ideal for relating behavioral and neural measures of ToM (Rice and Redcay, 2015).

Given the large range and variability of SCQ scores, why isn’t this measure more sensitive to other aspects of the functional response in ToM regions? In the previous study, proportion of correct responses on a ToM task was correlated with inter-region correlations, functional maturity, and response magnitude to a ToM event in the ToM network; the correlation with response magnitude remained significant when additionally controlling for age. In the current study, SCQ score was correlated with the response magnitude to one ToM event (described above), but uncorrelated with the other neural measures. One possible explanation for the overall weak relationship is that the SCQ measure is not optimal for measuring individual differences in social cognition that are relevant for these neural responses. The SCQ is a parent questionnaire comprised of Yes/No questions about their child’s social and communication skills (Rutter et al., 2003). Many questions ask parents to “focus on the time period between the child’s fourth and fifth birthday,” which could be challenging, especially for parents of the oldest participants (twelve year old children, requiring memories from eight years earlier). By contrast, the previous study used a publicly available ToM behavioral battery to measure ToM reasoning (https://osf.io/g5zpv/), which requires children to answer prediction and explanation questions that draw on multiple concepts in ToM (e.g., similar/diverse desires, true/false beliefs, knowledge access, moral blameworthiness, mistaken referents, non-literal speech). In a prior study, performance on this task correlated with response selectivity in right temporo-parietal junction (Gweon et al., 2012). Thus, variability in performance on the booklet task might more sensitively index individual differences in ToM than the SCQ. A second possible explanation is that the two measures were designed to be differentially sensitive to change with age: the ToM booklet measure aims to reflect developmental change with age in addition to individual differences in ToM (i.e., a “state” measure), while the SCQ was designed to be sensitive to stable individual differences (i.e., a “trait” measure), and includes items that ask about a child’s social cognitive abilities at various ages (as described above). A third possible explanation is that apparent deficits captured by the SCQ are not caused by differences in basic processing of social stimuli, as reflected by inter-region correlations and properties of the functional response in ToM brain regions (Kliemann et al., 2018). Instead, these deficits may be better captured by measures of other neural systems, like those underlying social motivation, or by measures of the interactions between different neural systems (Kennedy and Adolphs, 2012). In any case, it is clear that the behavioral task choice is important for measuring correlations between cognitive and neural measures. While it is difficult to constrain or tease apart specific social cognitive concepts used while viewing a movie stimulus, behavioral tasks can help to clarify the components of social cognitive reasoning that are captured by neural individual differences. Researchers should look to relevant tasks used in developmental literature (Decety et al., 2008; Gweon et al., 2012; Richardson et al., 2018; Sabbagh et al., 2009), and consider adapting tasks that reflect neural individual differences in adults (Kanske et al., 2015; Rice and Redcay, 2015).

The results of the current study provide several extensions of the previous study. First, while the current study generally replicated evidence for developmental change with age, developmental trends were most apparent in a wide age range of children. For example, in the previous sample as well as in the current sample, within-network correlations showed moderate (non-significant) developmental increases between ages five to twelve years. However, expanding the age range to include younger participants (as in the previous study) and older participants (as in the current study) revealed strong evidence for developmental change with age. Thus, measuring developmental change in ToM and pain brain regions may require large samples that utilize wide age ranges. One challenge for this kind of research is designing an experimental paradigm that is suited for such wide age ranges. Movie-viewing paradigms offer one promising solution to this challenge, as they are generally engaging for participants of many ages.

Second, while functional maturity (i.e., similarity to the average “adult” timecourse) was significantly correlated with the extent to which the ToM and pain brain regions were *anti-correlated* (as reported by the previous study), this measure was also significantly positively correlated with the extent to which brain regions *within* the ToM network were correlated. This pattern of results was also true in the previous study, when analyzing inter-region correlations in raw timecourses (see Supplementary Materials). Thus, it is likely that both within-network and across-network correlations contribute to the maturity of the functional response in ToM and Pain brain regions.

A key goal of this study was to characterize the nature of the link between the stimulus-driven timecourse in ToM and pain brain regions, and the inter-region correlations within and between these two networks. Inter-region correlations in ToM and pain brain regions were measured during rest, in order to determine whether the link between functional maturity and inter-region correlations was specific to stimulus-driven responses, or reflective of intrinsic properties of these two networks. In the current dataset, the responses in the ToM and pain brain regions showed high within-network correlations and negative across-network correlations at rest, and inter-region correlations measured at rest were significantly positively correlated with those measured during naturalistic movie-viewing. Interestingly, within-network correlations were *higher* in absence of stimuli, relative to during movie-viewing. Previous studies have suggested that the extent of the correlation within- and across- brain regions varies by task. However, evidence regarding the direction of the effect of tasks on intrinsic correlations is mixed. Some studies report enhanced inter-region correlations during tasks, relative to rest (Vanderwal et al., 2017). Others, like this study, show reduced inter-region correlations during task, relative to rest (Betti et al., 2013; Emerson et al., 2015; Greene et al., 2018). One possibility is that the direction of this effect depends on the relevance and specificity of the content of the stimulus for the functional regions examined (Dixon et al., 2017; Gratton et al., 2016).

Critically, the extent to which ToM and Pain networks were functionally dissociated (i.e., anti-correlated) *only during movie-viewing* predicted the functional maturity of the responses in these networks. This is consistent with previous evidence that the correlations between “default mode” brain regions are altered during narrative processing (Simony et al., 2016). Despite the high correlation between the two measures (Gratton et al., 2018), the differences between inter-region correlations measured during a task versus at rest are apparently relevant for relating these measures to functional properties of the neural response. Interestingly, the movie stimulus appeared to reduce variance in inter-region correlations. The remaining variability in inter-region correlations during relevant functional tasks or movies may best capture relevant individual differences in functional response maturity (Finn et al., 2017).

Given the similarity of inter-region correlations measured during movie-viewing and during rest, pediatric imaging studies of children (who are old enough to be awake/engaged by movies) that aim to characterize functional responses should strongly consider using movie-viewing paradigms. Functional imaging of pediatric samples is notoriously effortful and costly, in particular because it is difficult to completely prevent participant motion. The current study finds that participant attrition and motion is reduced in movie-viewing paradigms relative to resting state scans (see also (Raschle et al., 2009; Vanderwal et al., 2015)), and that paradigms that evoke preferential functional responses are more sensitive to the maturity of functionally selective brain regions. Thus, movie-viewing paradigms may enable researchers to conduct previously intractable pediatric studies with less subject attrition and cost. To date, fMRI studies using movie-viewing paradigms have measured responses in ToM brain regions in children who pass and fail false-belief tasks (ages 3–5 years (Richardson et al., 2018)), and in letter- and number-selective brain regions in children who are learning to read and do math (ages 4–6 years (Cantlon and Li, 2013)). Among adults, naturalistic movie-viewing or story-listening paradigms have been used to study face- and scene-selective brain regions (Hasson, 2004), and networks of brain regions recruited to process language or complete cognitively challenging tasks (Blank et al., 2014; Paunov et al., 2017) (in addition to social brain regions: (Hasson, 2004; Jääskeläinen et al., 2008; Lerner et al., 2011; Nummenmaa et al., 2012; Richardson et al., 2018; Wilson et al., 2007)). Thus, using movies to measure functional responses in children is a promising avenue for future pediatric research. Future directions include conducting longitudinal or training studies to clarify the causal order of development of functional and intrinsic network properties in ToM and pain brain regions (Gabard-Durnam et al., 2016; Mackey et al., 2013), and examining the relationship between functional and structural brain development (e.g., white matter tract development), and the relative contributions of each to cognitive change.

## 5 Conclusion

A key challenge for developmental cognitive neuroscience is to develop experiments that are feasible for children, replicable, and useful for relating brain development to cognitive change. This study used a publicly available fMRI dataset to provide confirmatory evidence for early signatures and developmental change in the cortical dissociation between regions that process information about others’ bodies (the “Pain Matrix”) and those that process information about others’ minds (the “Theory of Mind” network). In doing so, this study (1) suggests a replicable neural marker of social cognitive reasoning that is measureable across multiple movie-viewing-paradigms, and (2) clarifies the relationship between inter-region correlations during movie-viewing, “intrinsic” inter-region correlations present at rest, and functional responses in the developing brain. Further, the current study demonstrates the promise of naturalistic movie-viewing experiments for replicating results across research sites and samples, and for future studies of pediatric and clinical populations.

## Conflict of interest

The author declares no competing financial interests.
