# Supplementary material for: Development of brain networks for social functions: Confirmatory analyses in a large open source dataset
Source: Dev Cogn Neurosci. 2018 Nov 20;37:100598. doi: 10.1016/j.dcn.2018.11.002 (PMC6969289; doi:10.1016/j.dcn.2018.11.002)
Supplement: Supplementary file 1 [file mmc1.pdf]

Supplementary Materials

Possible Causes of Participant Attrition

The current study had high rates of participant attrition, relative to the prior study. This difference in attrition could plausibly be due to the inclusion of participants with clinical diagnoses, including a large number of individuals diagnosed with Attention-Deficit/Hyperactivity Disorder (ADHD) (see Supplementary Figure 2). Core features of ADHD are difficulties with attention and sustained focuses, and hyperactivity, i.e., excessive fidgeting or restlessness. These kinds of difficulties likely make staying still during an fMRI experiment challenging. By contrast, the prior study recruited participants with no known cognitive or neural disorders. The two samples also differed in IQ (see Supplementary Figure 1), with the current sample having a representative distribution of IQ, and the prior sample having more above average IQ scores.

The difference in attrition between studies could also plausibly be due to differences in experimental procedures. Both studies used a mock scanner session to acclimate children to the scanner environment. However, the prior study additionally used custom-made pediatric head coils. Smaller head coils reduce the possible amount of head motion. Richardson et al. (2018) additionally allowed children to hold a large stuffed animal during the scan, which, anecdotally, helped children to fidget with their hands and touch their face less, and helped some children to feel calm.

Reanalysis of Previous Study: 5 – 12 Year Old Participants Only

For the closest comparison of the results of the previous study to the current results, the previous sample was re-analyzed, excluding the three and four year old children.

Developmental Change in Inter-Region Correlations

In the previous study, evidence for developmental increases in within-network inter-region correlations depended on including the youngest children scanned (ages 3-4 years old); within-network correlations were not significantly correlated with age among the 5-12 year old children (spearman partial correlations including motion as covariate: ToM: $r_s(88)=-.03$, p=.75; Pain: $r_s(88)=.08$, p=.45). However, evidence for decreases in across-network correlations with age remained significant in 5-12 year old children ($r_s(88)=-.35$, p=.0007).

Functional Maturity

In the previous study, "functional maturity" (i.e., similarity to responses in adults) among 5 – 12 year olds increased with age in both networks, and was significantly positively correlated with the extent to which the ToM and Pain networks were *anti-correlated* during the task (controlling for age, within-network correlations, and motion). That is, children who had more anti-correlated ToM and Pain response timecourses also had timecourses that were more similar to adult participants. These results remained significant among 5 – 12 year old participants (excluding 3 – 4 year olds): functional maturity increased with age (spearman partial correlations including motion as a covariate: ToM: $r_s(91)=.38$, p=.0002; Pain: $r_s(91)=.48$, $p=1.5\times10^{-6}$) and was predicted by the anti-correlation of responses in the two networks (linear regressions testing for effects of across-network correlation, within-network correlation, age, and motion on functional maturity measure (n=91): ToM: effect of across-network correlation: b=-.7, t=-7.3, $p=1.4\times10^{-10}$, effect of within-ToM correlation: b=.02, t=.28, p=.007, NS effect of age: b=.13, t=1.6, p=.10,

effect of motion: b=-1.7, t=-2.1, p=.04; Pain: effect of across-network correlation: b=-.69, t=-6.7, $p=1.7x10^{-9}$, NS effect of within-Pain correlation: b=.22, t=2.3, p=.03, effect of age: b=.22, t=2.6, p=.01, NS effect of motion: b=-.10, t=-1.4, p=.17). However, these results show that within-network correlations also positively predicted functional maturity.

Reanalysis of Previous Study: 5 – 12 Year Olds Only & M1 Timecourse Regression
In the previous study, primary inter-region correlation analyses were conducted on residual response timecourses, after regressing out the average bilateral primary motor (M1) cortex timecourse. Inter-region correlation analyses of the raw timecourses were included as supplementary analyses. In the current study, inter-region correlation analyses were conducted on the raw timecourses (without regressing out the M1 timecourse). The re-analysis of the original sample, excluding three and four year old participants, using residual timecourses (with M1 regressed out) are reported below.

Developmental Change in Inter-Region Correlations
As in the analyses of the raw timecourse (above), evidence for developmental increases in within-network inter-region correlations in the previous dataset depended on including the youngest children scanned (ages 3-4 years old); within-network correlations did not change with age among the 5-12 year old children (spearman partial correlations including motion as covariate: ToM: $r_s(88)=-.03$, p=.75; Pain: $r_s(88)=.13$, p=.21). However, evidence for decreases in across-network correlations with age remained significant in 5-12 year old children ($r_s(88)=-.35$, p=.0007).

Functional Maturity Analysis
The previous study suggested that across-network inter-region correlations (and not within-network inter-region correlations) were significantly correlated with functional maturity. This result remains the same in an analysis of 5 – 12 year old participants only (linear regressions testing for effects of across-network correlation, within-network correlation, age, and motion on functional maturity measure (n=91): ToM: effect of across-network correlation: b=-.5, t=-6.3, $p=1.1x10^{-8}$, NS effect of within-ToM correlation: b=.02, t=.22, p=.82, effect of age: b=.2, t=2.1, p=.04, effect of motion: b=-.2, t=-2.6, p=.01; Pain: effect of across-network correlation: b=-.57, t=-7.1, $p=3.1x10^{-10}$, NS effect of within-Pain correlation: b=.09, t=1.2, p=.22, effect of age: b=.3, t=3.3, p=.001, NS effect of motion: b=-.08, t=-1.0, p=.3).

Lasso Regressions: Effects of Inter-Region Correlations on Functional Maturity
Lasso regressions were conducted in the low/matched-motion subset of participants (n=106) to simultaneously test for correlations between inter-region correlations measured at rest and measured during movie viewing and functional maturity, per network. The predictors included in these regressions were: across-TP-movie, across-TP-rest, within-[ToM or Pain]-movie, within-[ToM or Pain]-rest, age, motion (average number of artifact timepoints across the movie and resting state scans). As determined by minimizing Mallow's Cp (Mallows, 1973), in the ToM network, functional maturity was best predicted by a model that included across-TP-movie (b=-.07), wi-ToM-movie (b=.30), motion (b=-.01), and within-ToM-rest (-.24) predictors, in that order. In the Pain network, functional maturity was best predicted by a model that included all predictors, in the following order: across-TP-movie (b=-.09), wi-Pain-movie (b=.23), age (b=.008), across-TP-rest (b=.06), motion (b=-.004), within-Pain-rest (b=.18). Note that the sign

flip on the within-ToM-rest predictor is likely reflective of multi-collinearity among the predictors (Yoo et al., 2014).

Supplementary Figure 1

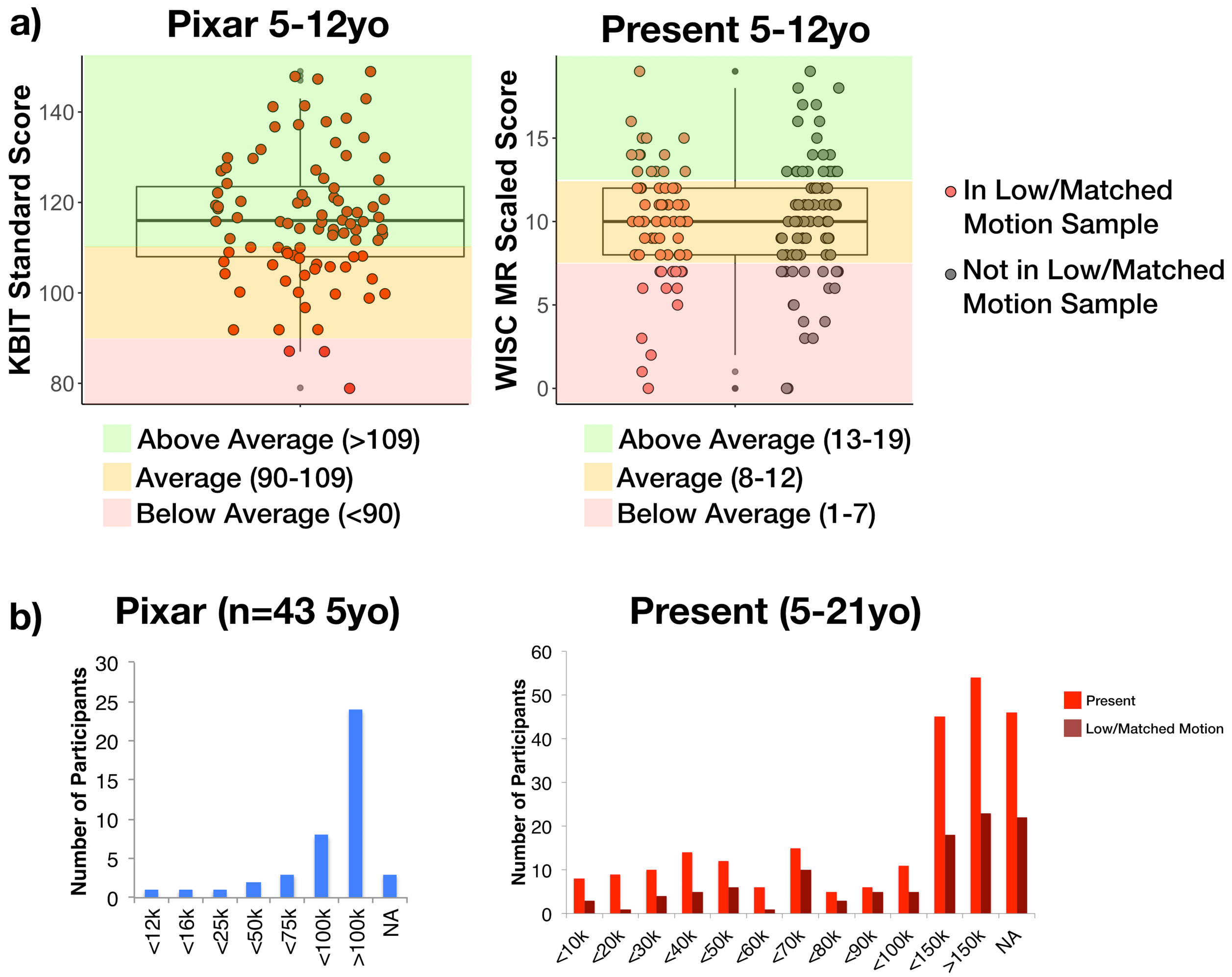

Supplementary Figure 1. Sample Characteristics. a) Performance on standardized test of non-verbal IQ in current and prior sample. Left: Child Participants in the prior study completed the non-verbal matrix reasoning component of the KBIT-II (A. S. Kaufman, 1997). Boxplot shows standardized scores; color bars indicate qualitative descriptors of performance. Overall, participants had higher than average IQ: the body of the boxplot falls mostly within the "above average" range. **Right:** Child participants in the current study completed the matrix reasoning subtest of the WISC-V (A. S. Kaufman, Raiford, & Coalson, 2015). Boxplot shows scaled scores; color bars indicate qualitative descriptors of performance. This sample is more representative in that the body of the boxplot falls within the "average" range, rather than in the "above average" range. **b) Annual household income in current and prior sample.** Both plots show the number of participants (y-axis) who resided households with varying amounts of annual income (x-axis). The x-axis varies slightly across studies due to differences in the socioeconomic status questionnaire used; the prior study used a custom questionnaire, and the current study used the Financial Support Questionnaire (FSQ). Both studies show that participants from lower-income households are under-represented. The current sample has a higher number of participants from lower-income households, but the distribution of household income is relatively similar across studies. Note that the data plotted for the prior study includes the five-year-old children only (n=43); the remaining participants were not asked to complete the questionnaire.

Supplementary Figure 2

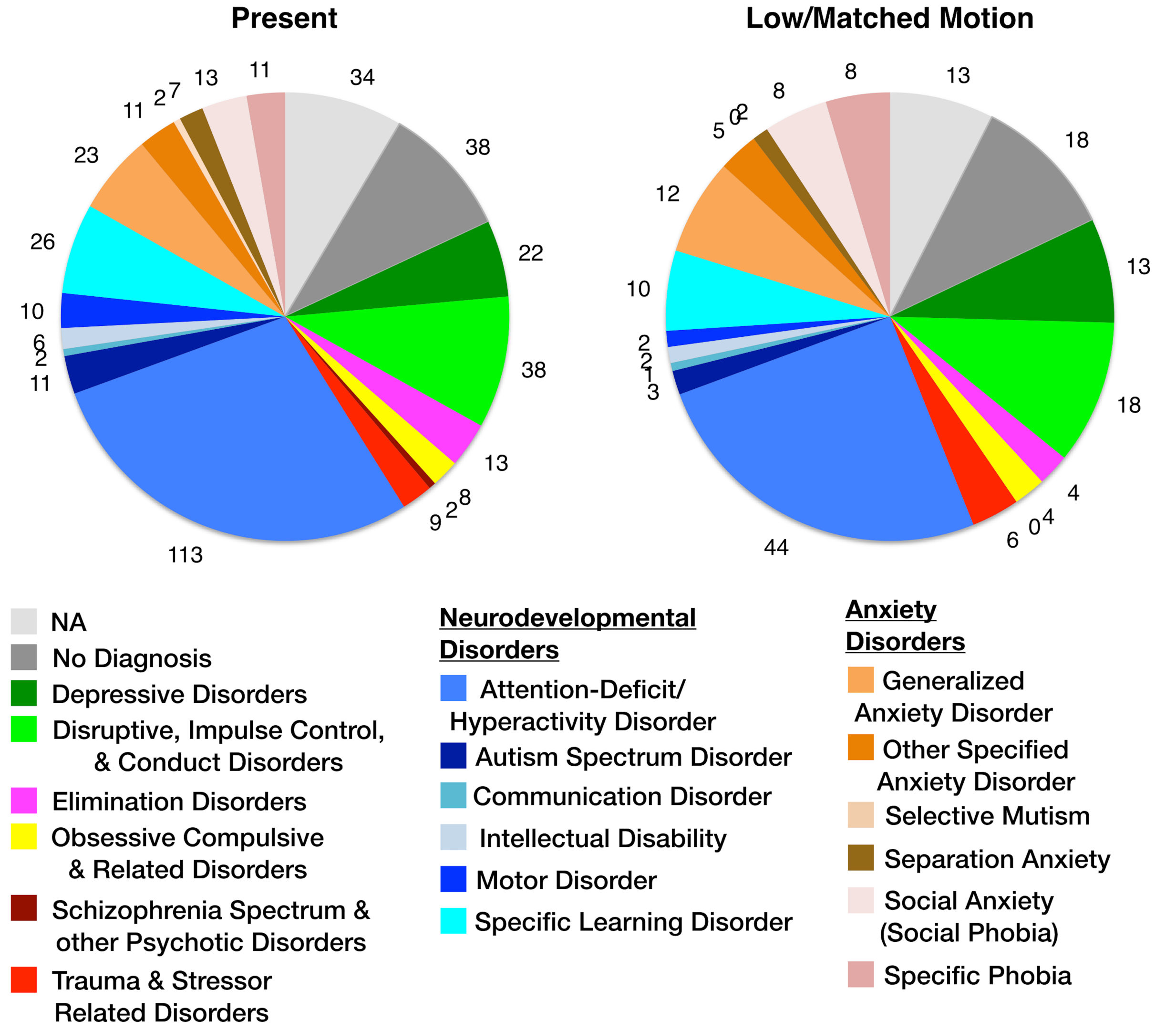

Supplementary Figure 2. Clinical Diagnoses. Unlike the prior study, in which all participants had no known cognitive or neurological disorders, many participants in the current sample had a clinical diagnosis at the time of participation (Alexander et al., 2017). The numbers outside of the pie chart indicate the number of individuals with each diagnosis. Note that an individual can have multiple diagnoses; all diagnoses that did not need confirmation or further evaluation are included. The distribution of clinical diagnoses was similar across the full sample (left) and the low/matched motion subset of data (right). The modal diagnosis was Attention-Deficit/Hyperactivity Disorder (medium blue; n=113 in "The Present"; n=44 in low/matched motion subset). The plots show the "Consensus Diagnosis" data, which were based on the results of the K-SADS interview (J. Kaufman et al., 1997), based on DSM-IV (1994) 4th ed. criteria. The K-SADS was administered to both a participant and his/her parent or caregiver; a research clinician then determined the set of diagnoses that were consistent between the child and parent/caregiver interviews.

Supplementary Figure 3

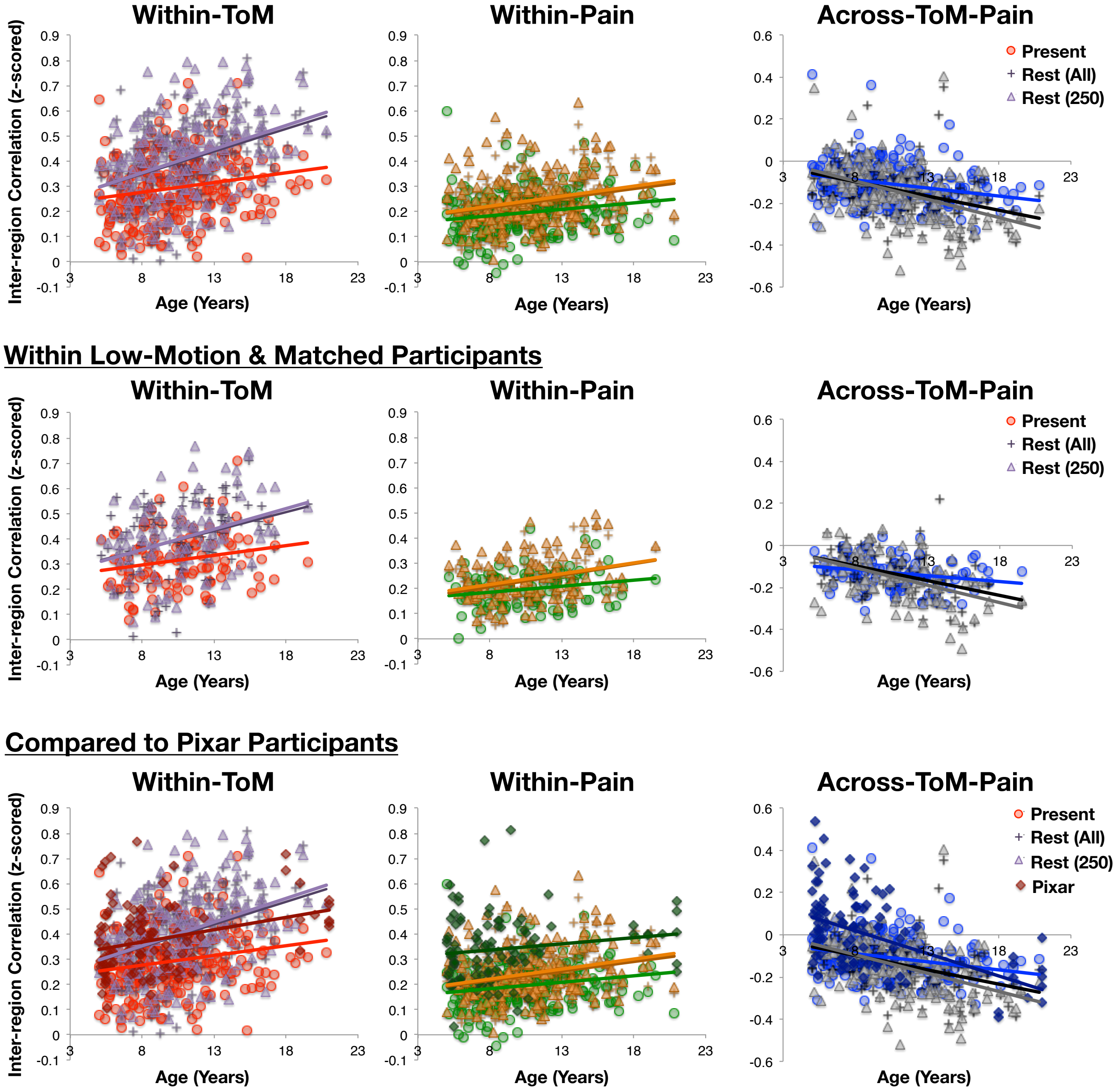

Supplementary Figure 3. Inter-region Correlations during Movie Viewing and at Rest. All scatter plots show z-scored inter-region correlations (y-axis) during movie viewing/at rest, by age (x-axis) within the ToM network (left, red (movie)/purple (rest)), within the Pain network (middle, green (movie)/orange (rest)), and across the ToM-Pain networks (right, blue (movie)/grey (rest)). Circles show inter-region correlations as measured during Jacob Frey's "The Present" (Frey, 2014); triangles show inter-region correlations as measured during the length-matched resting state scan (250 TRs; included in all main analyses); plus signs show inter-region correlations as measuring during the full resting state scan (750 TRs). The top row includes data from all participants (n=238 for "The Present", n=200 for resting). The middle row includes data from the low/matched motion subset of participants (n=106). The bottom row shows all data from the current sample (identical to the top row) as well as data from the prior study (Richardson, Lisandrelli, Riobueno-Naylor, & Saxe, 2018) ("Pixar", diamond data points). Pixar participants include n=91 children (5 – 12 years old) and n=11 adults (18-21 years old). Older adults (n=22, ages 22-39 years) and younger children (n=31 3 – 4 year olds) were excluded from these plots in order to better match the age range of the current sample.

Supplementary Figure 4

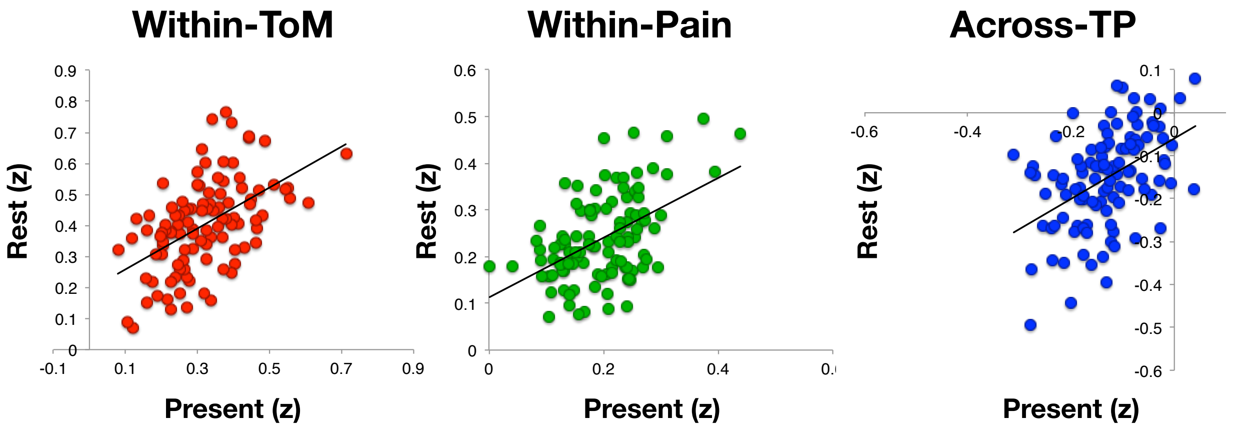

Supplementary Figure 4. Inter-region Correlations during Movie Viewing and at Rest are Correlated. Scatter plots show z-scored inter-region correlations as measured during "The Present" (x-axis), by those measured during rest (y-axis) within the ToM network (left, red), within the Pain network (middle, green), and across the ToM-Pain networks (right, blue), in the low/matched motion subset of participants (n=106).

Supplementary Figure 5

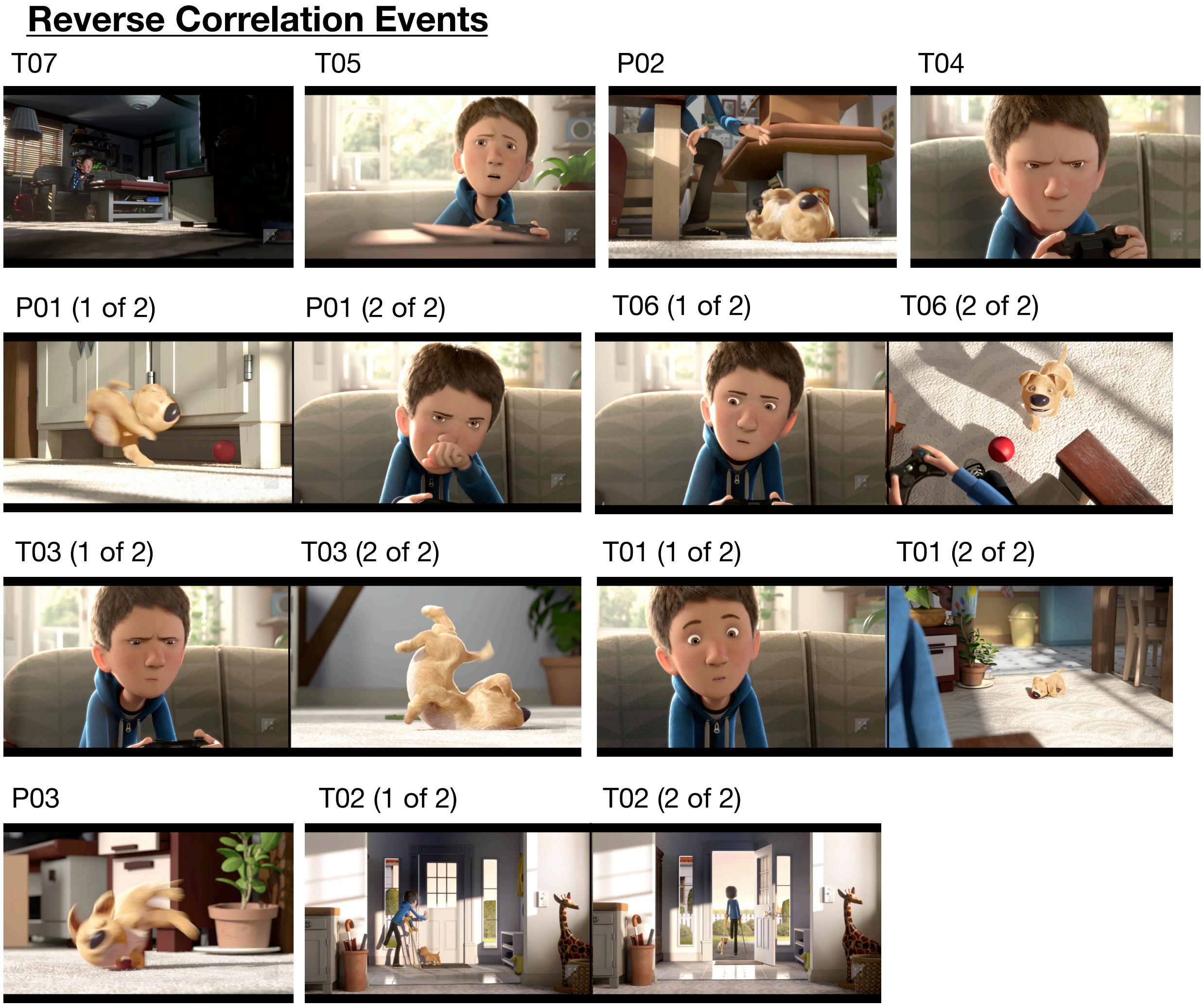

Supplementary Figure 5. Reverse Correlation Events. Thumbnail images of each event identified by the reverse correlation analysis of the timecourse of response during “The Present” (Frey, 2014) in adolescent and young adult participants (ages 13-20 years, n=55). See Supplementary Table 1 for descriptions and timing information. Thumbnail images used with permission from Jacob Frey.

Supplementary Figure 6

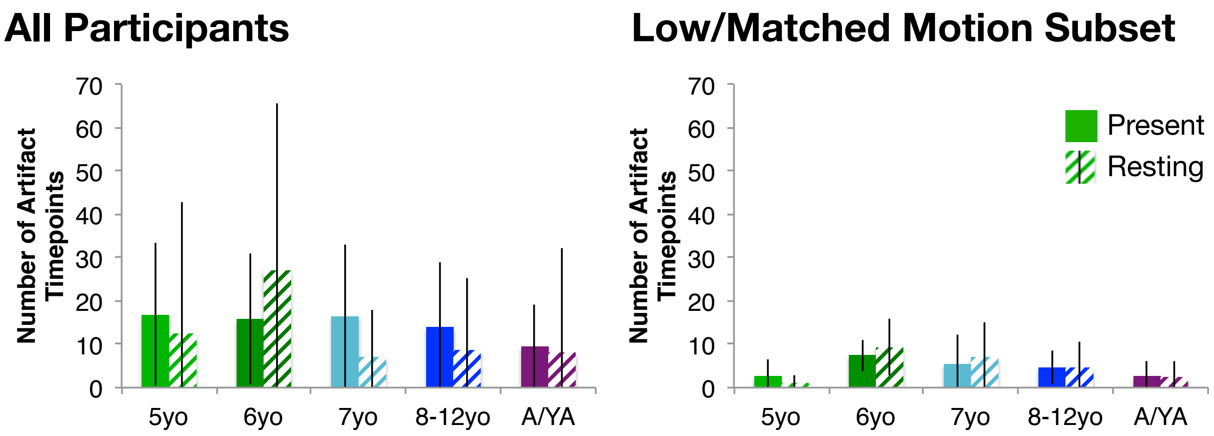

Supplementary Figure 6. Amount of Motion during FMRI Scans. Plots show mean number of artifact timepoints (>2mm motion, > 3 standard deviation from average global signal) per age group during "The Present" (solid bars) and during the resting state scan (striped bars). The plot on the left includes all participants (n=241); the plot on the right includes the low/matched motion subset of participants (n=106). Error bars show standard deviation from the mean.

Supplementary Table 1

	Event	Time in Movie (m:s:ms)	Duration (s)	Peak Timepoint (TR)	Description
ToM Events	T01	2:13:80 - 2:21:00	140	175	Boy is softening, seems conflicted. Watches puppy.
	T02	2:58:60 - 3:10:60	190.4	238	Boy heads towards door. It becomes clear that he is missing a leg, like the puppy. Puppy and boy go outside together.
	T03	1:52:20 - 1:56:20	117.6	147	Boy looks annoyed; puppy heads towards ball in box.
	T04	1:21:00 - 1:25:00	86.4	108	Boy looks annoyed; puppy looks around.
	T05	0:41:80 - 0:46:60	45.6	57	[Mom just told boy to open present]. Boy says "for me?" and looks at box.
	T06	1:40:20 - 1:44:20	105.6	132	Boy notices ball; puppy approaches boy expectantly.
	T07	0:10:60 - 0:14:60	17.6	22	Boy playing video game.
Pain Events	P01	1:31:40 - 1:39:40	99.2	124	Puppy slams into cabinet. Boy wipes nose.
	P02	1:04:20 - 1:09:80	72	90	Boy notices missing leg and tosses puppy to floor. Boy hits present box.
	P03	2:24:20 - 2:32:20	154.4	193	Puppy carries ball over to boy, fumbling a bit because of his missing leg.

Supplementary Table 1. Reverse Correlation Events. Table includes the name, time, duration, peak timepoint, and description of each event identified by the reverse correlation analysis of the timecourse of response during "The Present" in adolescent/young adult participants (n=55). Event name indicates the rank of the event (T01 is the event with the highest peak magnitude of response, T02 the second highest, etc.). See Supplementary Figure 5 for thumbnail images of each event.

References

Alexander, L. M., Escalera, J., Ai, L., Andreotti, C., Febre, K., & Mangone, A. (2017). The healthy brain network biobank: an open resource for transdiagnostic research in pediatric mental health and learning disorders. bioRxiv. *bioRxiv*.

American Psychiatric Association (2000). *Diagnostic and statistical manual of mental disorders* (4th. ed). Washington, D.C.

Frey, J. *The Present* [Motion Picture]. 3:23 min. (2014).

Kaufman, A. S. (1997). KBIT-2: Kaufman Brief Intelligence Test. Minneapolis, MN: NCS Pearson.

Kaufman, A. S., Raiford, S. E., & Coalson, D. L. (2015). Intelligent testing with the WISC-V. John Wiley & Sons.

Kaufman, J., Birmaher, B., Brent, D., Rao, U., Flynn, C., Moreci, P., et al. (1997). Schedule for affective disorders and schizophrenia for school-age children-present and lifetime version (K-SADS-PL): initial reliability and validity data. *Journal of the American Academy of Child & Adolescent Psychiatry*, *36*(7), 980–988.

Mallows, C. L. (1973). Some comments on C p. *Technometrics*, *15*(4), 661–675.

Richardson, H., Lisandrelli, G., Riobueno-Naylor, A., & Saxe, R. (2018). Development of the social brain from age three to twelve years. *Nature Communications*, *9*(1), 1027.

Yoo, W., Mayberry, R., Bae, S., Singh, K., He, Q. P., & Lillard, J. W., Jr. (2014). A study of effects of multicollinearity in the multivariable analysis. *International Journal of Applied Science and Technology*, *4*(5), 9.
